# Fulvic acid increases forage legume growth inducing preferential up-regulation of nodulation and signalling-related genes

**DOI:** 10.1093/jxb/eraa283

**Published:** 2020-06-30

**Authors:** Nicola M Capstaff, Freddie Morrison, Jitender Cheema, Paul Brett, Lionel Hill, Juan C Muñoz-García, Yaroslav Z Khimyak, Claire Domoney, Anthony J Miller

**Affiliations:** 1 Department of Metabolic Biology, John Innes Centre, Norwich Research Park, Norwich, UK; 2 School of Pharmacy, University of East Anglia, Norwich Research Park, Norwich, UK; 3 University of Warwick, UK

**Keywords:** Forage crops, fulvic acid, humic substances, *Medicago sativa*, nodulation, transcriptomic analysis, yield

## Abstract

The use of potential biostimulants is of broad interest in plant science for improving yields. The application of a humic derivative called fulvic acid (FA) may improve forage crop production. FA is an uncharacterized mixture of chemicals and, although it has been reported to increase growth parameters in many species including legumes, its mode of action remains unclear. Previous studies of the action of FA have lacked appropriate controls, and few have included field trials. Here we report yield increases due to FA application in three European *Medicago sativa* cultivars, in studies which include the appropriate nutritional controls which hitherto have not been used. No significant growth stimulation was seen after FA treatment in grass species in this study at the treatment rate tested. Direct application to bacteria increased *Rhizobium* growth and, in *M. sativa* trials, root nodulation was stimulated. RNA transcriptional analysis of FA-treated plants revealed up-regulation of many important early nodulation signalling genes after only 3 d. Experiments in plate, glasshouse, and field environments showed yield increases, providing substantial evidence for the use of FA to benefit *M. sativa* forage production.

## Teaser

Fulvic acid treatment increases yield and nodulation in *Medicago sativa*, in glasshouse and field experiments. *De novo* transcriptome analysis shows the up-regulation of early nodulation genes in response to fulvic acid.

## Introduction

Forage grasslands represent 26% of global land area and 70% of agricultural land (FAO and IFIF, 2010). In temperate climates, forage crops are cultivated and these are usually grasses (*Poaceae*) or herbaceous legumes (*Fabaceae*). The globally important legume lucerne or alfalfa (*Medicago sativa*) is of prominence in temperate forage production. For forage growers, increasing the crop’s yield is a primary focus, and new management practices to maintain or increase growth with lower nitrogen (N) inputs are needed. The application of a humic substance- (HS) derived biostimulant called fulvic acid (FA) may improve forage crop production.

Extractable HS fractions are considered to be key soil components, and their complex composition may be responsible for facilitating many complex chemical reactions in soil systems (Sutton and Sposito, 2005; Canellas *et al.*, 2010; Trevisan *et al.*, 2010*a*; Lamar *et al.*, 2013). Identification of the specific effects of HS requires the use of well-structured, specific methods. Research on FA is often limited by chemical characterization and frequently uses samples which are not easily replicable, because their source is not unique (Pandeya *et al.*, 1998). This makes designing appropriate controls for experiments difficult. Many studies often rely on a ‘no application’ or ‘water treatment’ as controls to determine the potential biostimulant effect of FA on a plant (Calvo *et al.*, 2014). In the model plant *Arabidopsis thaliana* and many cereal crops, HS has been shown to have effects on plant growth including increased root growth, improved nutrient uptake and yield under stress and control conditions, and enhanced access to metals (Pandeya *et al.*, 1998; Zhimang *et al.*, 2001; Pinto *et al.*, 2004; Dobbss *et al.*, 2007; García *et al.*, 2012; Vaccaro *et al.*, 2015; Zhang *et al.*, 2015; Bezuglova *et al.*, 2017). A study of particular importance to forage crops is that of the legumes, soybean (*Glycine max*), peanut (*Arachis hypogaea*), and clover (*Trifolium vesiculosum*) (Tan and Tantiwiramanond, 1983). This study showed that a sand growth medium supplemented with FA reduced the number of nodules whilst increasing the nodule weight in a dose-dependent manner. Application of HS to *Pisum sativum* also increased root nodulation and mycorrhizal colonization (Maji *et al.*, 2017). If such increases were able to improve N fixation in legumes, then this could increase the N storage of the vegetative tissue and perhaps the protein content. The important forage crop *M. sativa* has been found to increase in vegetative biomass after FA application but with variable responses (Little et al., 2013, 2014). Another study linked bulk soluble HS fractions to increased biomass of *M. sativa* and, moreover, nodulation with stimulated *Sinorhizobium* growth, but this study did not include nutritional controls and compared HS application with no addition (Xu *et al.*, 2018). Studies using various HSs including FAs have been carried out in other important legumes and forage grasses (Verlinden *et al.*, 2010; Aydin *et al.*, 2012; Traversa *et al.*, 2014; Daneshvar Hakimi Maibodi *et al.*, 2015; Chang *et al.*, 2016); however, again, no nutritional controls were used. Clearly, more detailed studies are required to fully assess the effect of FA on forage crops.

In recent years, RNA-sequencing (RNA-seq) has transformed from an exclusive tool used in discrete studies (Wang *et al.*, 2009; Marguerat and Bähler, 2010) to a critical technique accessible for many projects to investigate phenotypic changes occurring in specific conditions (Costa-Silva *et al.*, 2017). Changes in transcriptional expression in plants following stimulus, stress, or treatment can reveal the downstream signalling and metabolic changes that cause a phenotype. RNA-seq is an incredibly robust and sensitive tool (Mortazavi *et al.*, 2008; ‘t Hoen *et al.*, 2008; Martin *et al.*, 2013; Conesa *et al.*, 2016), providing a wealth of data that can provide an understanding of the underlying mechanisms underpinning a specific treatment.

Although a potential link between HS application and increased biomass and legume root nodulation has been demonstrated, the mechanism for the condition remains unknown. Previous studies have suggested wide-ranging modes of action for this biostimulant, including a hormone-like response by plants following HS addition (Nardi *et al.*, 1994; Russell *et al.*, 2006; Trevisan et al., 2010*a*, b; Canellas and Olivares, 2014), but there is a lack of transcriptional evidence to support this idea. Therefore, we have investigated the transcriptional changes that may occur in plants following FA treatment in both shoot and root tissues using RNA-seq analysis.

Two commercial FA formulations were tested in a range of important temperate forage crops including legumes, with *M. sativa* cultivars showing a stimulatory response to the application. In order to include appropriate nutritional controls for FA, chemical analyses of the commercial products were carried out. Treatments were first assessed in the glasshouse and controlled-environment room to establish growth effects on crops, and in *M. sativa* to establish a nodulation phenotype. Transcriptional changes were investigated for one FA treatment compared with its nutritional control, with *de novo* assembly and annotation of RNA-seq data, designed to provide evidence for the mode of action of FA linked to yield increases. Field trials were implemented at UK forage grower sites with applications and management using industry standard practices. The aim was to identify if a change in management practice including FA treatments can increase yield in forage cultivation under conventional farming methods.

## Materials and methods

### Chemical analysis

Two FA materials were acquired, VitaLink Fulvic (sourced from Holland Hydroponics & Horticulture, UK) and MPXA (F.A.R.M. Co., California, USA). These were termed VFA and MFA for subsequent work. The soluble dry weight of each FA was determined, and the elemental composition of solutions for total N, C, and trace elements was measured using inductively coupled plasma-optical emission spectrometry (ICP-OES) and inductively coupled plasma mass spectrometry (ICP-MS), performed for VFA at Computational and Analytical Sciences, Rothamsted Research, Harpenden, UK and for MFA at Biological Services, UEA, Norwich, UK. Samples (0.01 g ml^–1^) were analysed by GC-MS [Agilent GC-MS Single Quad Mass Spectrometer (7890/5977), Agilent Technologies, California, USA] and run information was as follows: samples were derivatized with MSTFA (Sigma-Aldrich 394866) on an Ultra 2 column (19091B-102; length 25 m, internal diameter 0.2 mm, film 0.33 μm; Agilent Technologies); carrier gas hydrogen at a constant flow of 1.2 ml min^–1^; inlet temperature 250 °C; injection volume 1 µl; injection mode split—splitless (30:1); oven temperature initial temperature 170 °C with ramp 10 °C min^–1^ to 300 °C and hold at 300 °C for 5 min, with equilibration time 0.5 min and auxiliary temperature 250 °C; acquisition mode, SCAN between 50 *m/z* and 800 *m/z*. Data were acquired with Agilent Masshunter Qualitative Analysis (B.07.00), and peaks were identified with the NIST Atomic Spectra database (v14, National Institute of Standards and Technology, Maryland, USA) (Smith *et al.*, 2005; Guijas *et al.*, 2018; Linstrom and Mallard, 2018). Samples were run with standards to confirm contents. Data from this analysis are given in the Supplementary data at *JXB* online: ICP in Supplementary Tables S1 and S2; and GC-MS in Supplementary Figs S1 and S2. Data were used to produce elemental controls for FAs to use in plant and microbial assays, called VC for VFA and MC for MFA, as listed in Supplementary Tables S3 and S4.

NMR was carried out to elucidate the type and ratio of functional groups present in FAs. ^1^H-decoupled ^1^H-^13^C cross-polarization (CP) and CP single pulse (CPSP) solid-state NMR experiments were performed at 20 °C using a 7.05 T Bruker Avance III spectrometer equipped with a 4 mm triple resonance probe operating at frequencies of 300.1 MHz (^1^H) and 75.5 MHz (^13^C). Each sample was packed into a zirconia rotor, sealed using a kel-f drive cap, and spun at 12 kHz. A CP contact time of 1 ms and relaxation delay of 5 s were employed, with 90° pulses of 3.5 µs and 4.5 µs used for ^1^H and ^13^C, respectively. All spectra were referenced with respect to TMS (Sigma-Aldrich, 87920). Peak areas were obtained from the CPSP experiments (i.e. containing both rigid and mobile components) using the automatic integration tool of TopSpin 3.6.1. Subsequently, they were normalized to relative areas and grouped into different functional groups according to the expected chemical shift regions for soil organic matter (Mathers and Xu, 2003; Mathers *et al.*, 2007): alkyl C (0–50 ppm), methoxyl C (50–60 ppm), carbohydrate C (60–90 ppm), di-*O*-alkyl C (90–110 ppm), aryl C (110–142 ppm), phenolic C (142–160 ppm), and carbonyl C (160–200 ppm). Data from NMR analysis are available in Supplementary Figs S3 and S4.

### Plant growth conditions

Three cultivars of *M. sativa* were tested: cv. Daisy (DLF Forage Seeds, DK), cv. Luzelle [Oliver Seeds Ltd (bred by INRA/Agri-Obtentions, FR, 1993)], and cv. Gea (DLF Forage Seeds, DK). The forage grass *Lolium perenne* cv. AberMagic (bred by IBERS-ABY-S562-2016) was also included.

*Medicago sativa* seeds were scarified prior to sterilization with concentrated H_2_SO_4_ followed by six washes with sterilized deionized water (dH_2_O). Seeds were then sterilized with a 10% (v/v) sodium hypochlorite solution containing 0.05% (v/v) Triton X-100 (X100) followed by six dH_2_O washes. The final wash included nystatin 5 µg ml^–1^ (Sigma-Aldrich N6261) and amoxicillin 50 µg ml^–1^ (Sigma-Aldrich A8523), and was filter sterilized to reduce fungal or bacterial contamination. The seeds were imbibed in this solution at 30±1 rpm for 2 h at 4 °C, and the wash was replaced for a repeat imbibing period. *Lolium perenne* seeds were surface sterilized with 70% ethanol. All seeds were washed in dH_2_O and plated on water agar [3 g of agar (AGA03, Formedium Ltd Norfolk, UK) in 200 ml of dH_2_O]. Seeds plates were vernalized for 2 d at 4–6 °C before being transferred to a controlled-environment room with temperature at 23 °C and photoperiod of 16 h light (90 µmol m^–2^ s^–1^)/18 h dark. Plants were germinated before transplantation to a glasshouse.

### Additional vegetative growth experiments (larger screen and plate environment)

The details of two additional vegetative yield experiments with FA treatment are available in the Supplementary data. For all growth experiments, FA or elemental controls were applied at the FA manufacturer’s recommended rate (1% in distilled water, 10 ml applied to the pot soil surface). This dosage rate was also used in the field trials and is therefore agriculturally relevant. First, a full screen of forage crops grown in the glasshouse was undertaken for VFA and MFA in comparison with dH_2_O only (Supplementary Fig. S5). Secondly, an *M. sativa* cv. Daisy screen on plates (without transfer to soil), both with and without inoculation with *Sinorhizobium meliloti* was undertaken (see Supplementary Fig. S6)

### Counts of *Sinorhizobium* colony-forming units (CFU)

*Sinorhizobium meliloti* 1021, kindly provided by Anne Edwards (Metabolic Biology, John Innes Centre), was pre-incubated in 100 ml of TY medium for 2 d at 28 °C shaking at 200 rpm to full cell density (OD_600 nm_ ~2.5), and then diluted for exponentially growing cultures to inoculate flasks for OD_600 nm_=0.1. Treatment flasks of 100 ml of TY were set up as follows: NA, no addition; dH_2_O, 10 ml of dH_2_O; VFA/MFA, 10 ml of autoclaved 10% VFA or 5% MFA; VC/MC, 10 ml of 10% VC or 5% MC. Flasks were inoculated with 10 μl of the strain and incubated at 28 °C. At time points of 0, 1, 2, 3, and 4 d, dilutions from treatment flasks of 10^–1^ to 10^–10^ were taken in triplicate and 10 μl of diluted samples was spotted onto TY agar plates. Plates were incubated at 28 °C for 1 d until single colonies had formed in a dilution of ~20–200. Rhizobial cell density was calculated for dilution factor and total volume of culture. The whole experiment was repeated in triplicate.

### Statistical analyses

Statistical analyses of measurements across triplicate experiments were calculated in Excel® 2016, with *S*tudent’s *t*-test for one-tailed distribution with homoscedastic data run between VFA/VC and MFA/MC; *P*-value denoted with * <0.05, ** <0.01, and *** <0.001. Significance between treatments was shown with letters using one-way ANOVA with Tukey testing in GenStat® 18th Edition (VSN International). Graphs were designed in RStudio.

### RNA-seq plant material

Seeds of *M. sativa* cv. Daisy were sterilized and sown in full seed trays (36×22×6 cm) containing Church farm soil at a rate of 20 kg ha^–1^. Trays were watered every 3–4 d, and at day 12 were treated with autoclaved 1% VFA or 1% VC to the soil at the base of the plant; VFA was compared in transcriptome analysis with VC due to it large response in both greenhouse and field trials. Plants were sampled for RNA at days 12 and 15, referred to as day 0 and day 3, respectively, in subsequent analysis. For each sample, 10 biological replicates were pooled, with shoot and root tissue separated to provide three experimental replicates from three trays. Tissue was immediately frozen in liquid N_2_ and stored at –80 °C.

### RNA extraction and sequencing

Total RNA was extracted using the TRI Reagent (Sigma-Aldrich, 93289) method with phase separation using 1-bromo-3-chloropropane and precipitation with 400 μl of isopropanol and 400 μl of of high salt precipitation solution: 0.8 M sodium citrate and 1.2 M NaCl. After incubation for 5 min at ~23–26 °C, and centrifugation at 12 000 g at 4 °C for 15 min, the pellet was washed with 1.5 ml of 70% (v/v) ethanol. Samples were air-dried for 5 min, and contaminated DNA was removed using the RNase-Free DNase Set (QIAGEN Ltd 79254).

Samples were purified using the RNeasy MinElute Cleanup Kit (QIAGEN Ltd, 74204) and initial quality was checked (Supplementary Table S5). Samples were diluted to 50–500 ng μl^*–*1^.

Library construction [poly(A) mRNA] and sequencing were performed by Novogene (HK) Company Ltd (Hong Kong) using the Next® Ultra™ RNA Library Prep Kit (New England BioLabs Inc., E7530L) and sequenced on one lane of a HiSeq™ 2000 (Illumina, HWI-ST1276) in high output mode using 150 bp paired-end reads and V2 chemistry; the sequencing quality check is shown in Supplementary Table S6.

### Read alignment and differential expression analysis

*De novo* transcriptome assembly was performed with Trinity (Grabherr *et al.*, 2011), which used all samples generated. A total of 630 599 transcripts were preliminarily identified, including isoforms (Supplementary Table S7). BUSCO (Kriventseva *et al.*, 2015) was run to check benchmarking of the assembly using Universal Single-Copy Orthologs. Kallisto (Bray *et al.*, 2016) was used to align the assembly which is less subjective than ballgown mapping, providing both transcripts per million (TPM) and reads per kilobase million (RPKM) for subsequent analysis.

Differential gene expression was determined for shoot and root tissue independently using Degust (Powell, 2015) with all read alignments. Tissue samples were grouped into treatment and time point. Transcripts with both an absolute log fold change (FC) of 0.585 (1.5× FC) and a false discovery rate- (FDR) adjusted *P*-value (*q*-value) <0.05 were considered as differentially expressed (DE) (Supplementary Fig. S7). For every grouping of tissue samples, all three experimental replicates were required to fit these criteria, thus ensuring a very high benchmark. Differential expression was checked using the voom/Limma method (Law *et al.*, 2014) for logFC between treatments (VC and VFA) at both time points (0 d and 3 d). Differential expression was then checked for individual treatments between time points. To eliminate any differences caused by random chance or plant development changes over the 3 d time scale, transcripts that were DE based on VFA treatment alone were calculated by subtracting 0VC versus 0VFA and 0VC versus 3VC from 0VFA versus 3VFA (Supplementary Table S8).

### Gene Ontology (GO) term identification, functional annotation, and enrichment testing

Root DE transcripts were imported into the Blast2GO v1.4.4 program pipeline (Conesa *et al.*, 2005; Gotz et al., 2008) as FASTA contigs for functional annotation. DE transcripts were checked against NCBI’s non-redundant NR database (Pruitt *et al.*, 2005) with a BLAST expectation value cut-off of 1.0E^–3^, and hits excepted for no more than 20 sequences. Mapping was run with the EMBL-EBI InterPro library (Mitchell *et al.*, 2019) using amino acid mapping (Carbon *et al.*, 2009) with all families, domains, sites, and repeats available tested. Annotation of mapped results was run using Gene Ontology Annotation Version 2019 (Ashburner *et al.*, 2000; The Gene Ontology Consortium, 2019) with the strict parameters: annotation cut-off of 55; GO weight of 5 only; E-value-Hit-Factor restricted to 1.0E^–6^; Hit filter set to 500; and Evidence Codes weighted from 0.5 to 1 depending on depth of evidence (default software parameters). The inbuilt statistical wizard in Blast2GO was used to generate distribution graphs for sequences and hit species, shown in Supplementary Fig. S8. To quality-check, a manual BLAST algorithm was performed (Altschul et al., 1990, 1997; Camacho *et al.*, 2009) with the NCBI database (Benson et al., 2005, 2013; Zaretskaya *et al.*, 2008; NCBI Resource Coordinators, 2016) of the 20 most significantly up- or down-regulated genes. Any genes lacking a GO annotation through InterPro were checked for annotation in QuickGO (Binns *et al.*, 2009) and UniProt (UniProt Consortium, 2018) and added to the analysis; functional annotations can be found in Supplementary Table S9, with a graph representing the top 20 biological process GO terms shown in Supplementary Fig. S9.

To test for enrichment of different categories of *de novo M. sativa* DE transcripts relative to all expressed transcripts found in *M. truncatula* (as the closest relative), the PANTHER Classification System v14.1 was used (Thomas *et al.*, 2003; Mi *et al.*, 2010). GO-Slim graphs were generated for molecular function, biological process, and protein class, and then an over-representation test was performed using Fisher’s exact test (Thomas *et al.*, 2006); see Supplementary Table S10.

Following RNA-seq root analysis, quantitative reverse transcription–PCR (qRT–PCR) was used to measure expression of a subset of DE transcripts. A subset of seven genes was chosen to confirm with qRT–PCR. Primers were designed for genes using available *M. truncatula* sequences, and primers calculated to have 90–115% efficiencies were used in qRT–PCR.

Root RNA underwent cDNA synthesis using SuperScript™ II Reverse Transcriptase (Invitrogen™ 18064022, Life Technologies Ltd) with oligo(dT) (Invitrogen™ 18418012, Life Technologies Ltd), and qRT–PCR was performed with SYBR® Green JumpStart Taq ReadyMix (Sigma-Aldrich); details can be found in Supplementary Table S11. Expression of the genes of interest was calculated using the arithmetic mean Ct according to analysis with the 2^–ΔCT^ method (Livak and Schmittgen, 2001) using the reference gene *ACTIN2*, as expression variance was comparable across all samples. Mean relative expression was calculated for experimental replicates and compared with RPKM logFC of DE transcripts.

### Field trials

To assess if yield increases in *M. sativa* from VFA found in both plate and glasshouse experiments were applicable to growers, field trials were carried out over the 2017 and 2018 growing seasons. In 2017, trials were performed at Dengie Crops Ltd (Southminster, Essex, UK) with cvs Daisy and Fado. In 2018, the trials were at both Blankney Estates Ltd (Blankney, Lincolnshire, UK) and A Poucher and Sons (Bardney Dairies) Ltd (Market Rasan, Lincolnshire, UK) with the cvs Daisy and Gea, respectively.

Treatments to be tested were NA, dH_2_O, 1% VFA, and 1% VC. The individual experimental design of each plot is shown in Supplementary Fig. S10. Each trial contained 4–6 plots per treatment of areas 4–10 m^2^ with buffer zones between plots. As in the glasshouse trials, treatments were applied and, at 21 d post-treatment, samples were taken for vegetative biomass measurements using a randomized 625 cm^2^ area. Samples were also taken for protein and chlorophyll for 2018 trial plots and analysis carried out at British Chlorophyll Company Limited (Blankney Estates, Navenby, UK); protein was detected using the Kjeldahl method, and chlorophyll using a Soxhlet extraction (Supplementary Fig. S11).

## Results and discussion

### Analysis of FAs found varying chemical composition

FAs were analysed for their elemental content using a range of techniques, with results shown in Supplementary Tables S1 and S2 and Supplementary Figs S1–S4. The data show that the two FAs have very different compositions, despite being based on similar starting material and following the same extraction process. ^1^H-^13^C CPSP/MAS NMR experiments were carried out for the simultaneous quantification of mobile and immobile components of soils Supplementary Figs S3, S4), as it has been previously shown to be a powerful NMR methodology for the routine analysis of soils in the solid state. MFA showed a predominant presence of alkyl groups (~75%), followed by carbonyl (~16%), carbohydrate (~6%), and methoxyl (~3%) components (Supplementary Fig. S3). On the other hand, VFA is mostly made up of carbohydrates (~80%) and a small proportion of alkyl (~8%) components; it also contains some carbonyl (~7%), phenolic (~2%), and aryl (~2%) groups (Supplementary Fig. S4). Controlling the inorganic contents of FAs in elemental controls was most feasible (as shown in Supplementary Table S3 and S4), and with the organic contents compensated for with biologically available C in the form of sucrose. Controlling these contents where possible was imperative in determining an effect in plant assays and, by including nutritional controls, we can begin to determine if FAs are acting through a specific pathway with one or two active ingredients or as a nutritional additive. The lack of such controls in previous work may be the reason for the range of responses reported and perhaps for the plant hormone-like stimulatory response after HS application (Nardi *et al.*, 1994; Russell *et al.*, 2006; Trevisan et al., 2010*a*, *b*; Canellas and Olivares, 2014). Changes such as altered root architecture and uptake may be a nutritional effect. More recently, the standardization of HS analysis has been advocated, including the separation of C-containing groups (Lamar *et al.*, 2013; Zherebker *et al.*, 2018). Therefore, control solutions for plants and microbes were produced based on elemental analysis for MFA and VFA, termed MC and VC, respectively; a description is found in Supplementary Tables S3 and S4. Additional controls, as used in other studies, included dH_2_O and no application (NA).

### Fulvic acid increased growth of *Medicago sativa* and was not a nutritional effect

**Fig. 1.**
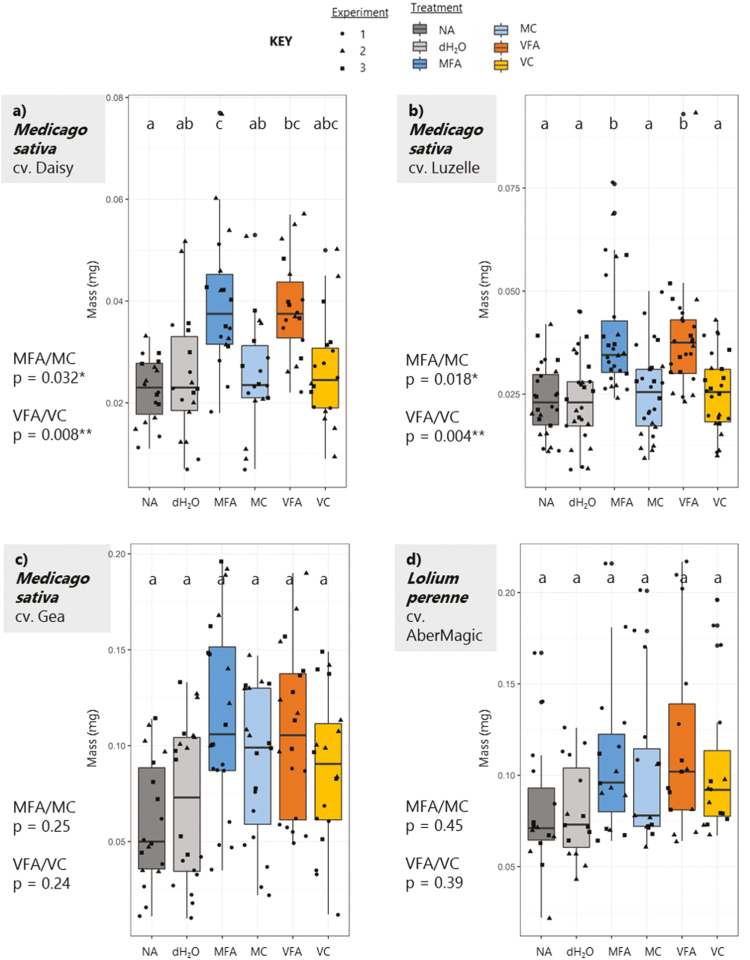
Vegetative biomass of *Medicago* cultivars and *Lolium* following treatment with fulvic acids or controls. Treatments were applied to seedlings at 7 d post-germination, and vegetative yields (DW in mg) were assessed at 21 d post-treatment. Treatments were: NA in dark grey; dH_2_O in grey; 0.5% MFA in blue; 0.5% MC in light blue; 1% VFA in orange; 1% VC in yellow. Three cultivars of *Medicago* were tested, cvs Daisy (a), Luzelle (b), and Gea (c). One cultivar of *Lolium* was tested, cv. AberMagic (d). Individual seedling biomass was measured for three independent experiments, as shown in black data points (Exp. 1=circles, Exp. 2=triangles, Exp. 3=squares). Box plots show variation across experiments. Multiple comparisons between treatments were conducted using a one-way ANOVA Tukey test shown with letters, and one-tailed Student’s *t*-tests were calculated for FAs and their controls, with *P*-value significance indicated on the left of the graphs.

Biomass yield assays in glasshouse conditions were carried out with *M. sativa* cultivars using applications of MFA and VFA alongside control solutions MC, VC, NA, and dH_2_O. Figure 1 shows vegetative biomass measurements recorded in three independent experiments for cvs Daisy, Luzelle, and Gea, alongside the grass *L. perenne* cv. AberMagic. Both cvs Daisy and Luzelle showed significantly increased vegetative growth after 3 weeks of FA treatment when compared with controls; cv. Gea also showed higher growth yields, but this increase was not statistically significant. This may be due to more interexperimental variation (as shown in individual sample points) due to the different time period when cv. Gea was tested during colder months in the glasshouse, with more rapid temperature fluctuations. The results in Fig. 1 demonstrate how application of FA can increase vegetative yield at very low concentration. This information supports existing indications of a potential yield effect of HS in *M. sativa* and similar forage legumes (Tan and Tantiwiramanond, 1983; Little et al., 2013, 2014; Xu *et al.*, 2018). Figure 1 also shows how the grass species *L. perenne* did not have increased vegetative biomass from FA application. Importantly, comparing the nutritional controls with dH_2_O treatments shows there was no significant nutritional component to the effect of FA application (see Fig. 1). Moreover, as shown in Supplementary Fig. S5 where FAs are tested with a larger screen of forage species, one can see how vegetative yield increases are found in legume species and not grasses when compared with dH_2_O only.

### Fulvic acid application caused an increased number of pink nodules

**Fig. 2.**
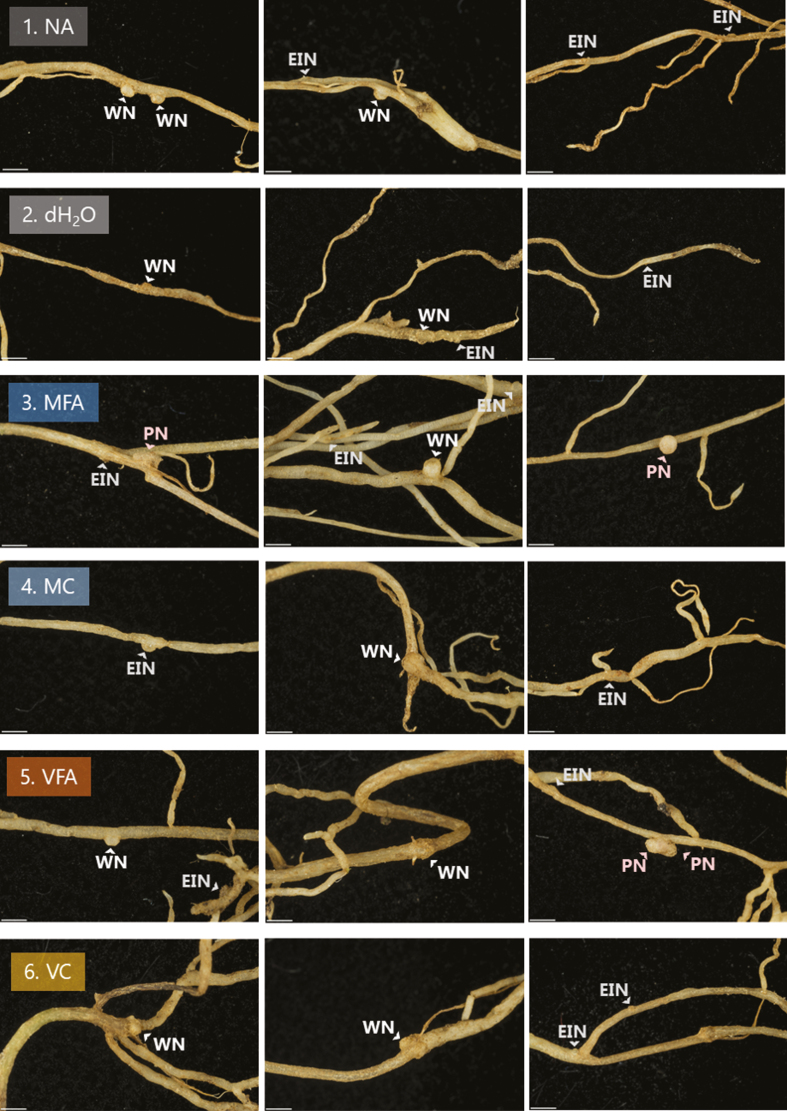
*Medicago sativa* cv. Daisy nodules following treatment with fulvic acids or controls. Treatments were applied to seedlings at 7 d post-germination and photographs were taken at 21 d post-treatment. Treatments were: 1. NA in dark grey; 2. dH_2_O in grey; 3. 0.5% MFA in blue; 4. 0.5% MC in light blue; 5. 1% VFA in orange; 6. 1% VC in yellow. Nodules are indicated as either early initiating nodules (EIN), white nodules (WN), or pink nodules (PN). Only white and pink nodules were counted as true nodules for this analysis. Scale included is 1 mm.

Yield in legumes is known to be affected by the degree of root nodulation by symbiosis with *Rhizobium*, including *Sinorhizobium*. The numbers of nodules were investigated in cvs Daisy and Luzelle, with counts performed on plants grown in both FA and control conditions. Figure 2 shows a representative visual scoring of cv. Daisy nodules for each treatment. This includes labelling of early stage initiating nodules (EINs), established white nodules (WNs), or mature pink nodules (PNs). Only WNs and PNs are included in counts.

**Fig. 3.**
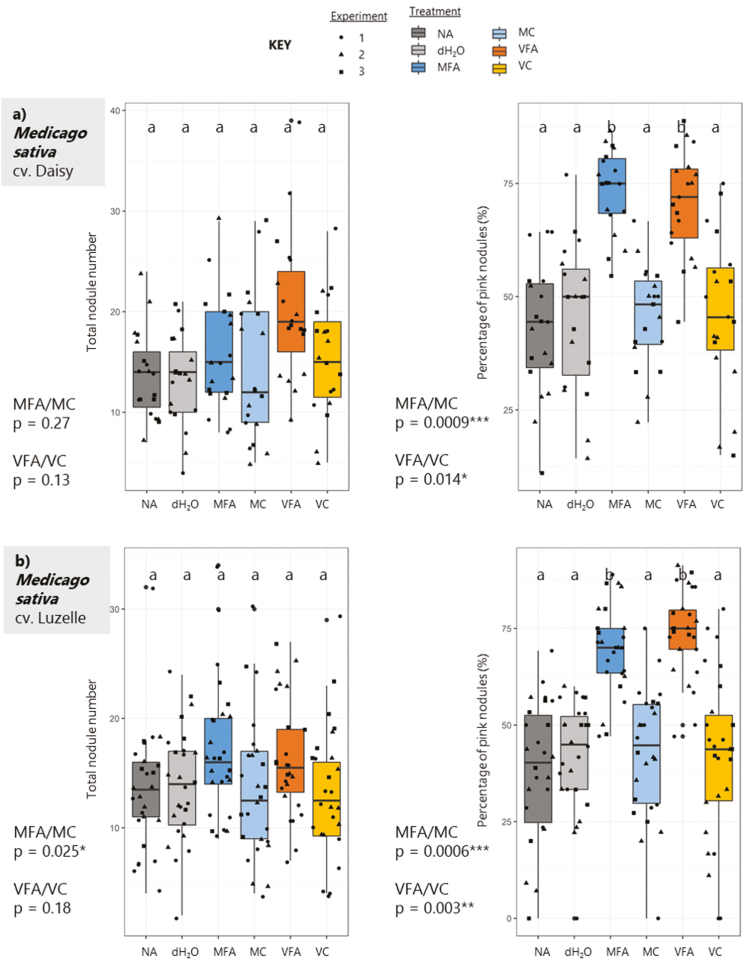
Nodulation counts of two *Medicago sativa* cultivars following treatment with fulvic acids or controls. Treatments were applied to seedlings at 7 d post-germination and nodules were counted at 21 d post-treatment. Treatments were: NA in dark grey; dH_2_O in grey; 0.5% MFA in blue; 0.5% MC in light blue; 1% VFA in orange; 1% VC in yellow. Two cultivars of *Medicago* were tested, cv. Daisy (a) and cv. Luzelle (b). Individual seedling nodules were counted for three independent experiments, as shown in black data points (Exp. 1=circles, Exp. 2=triangles, Exp. 3=squares). Box plots show variation across experiments. Multiple comparisons between treatments were conducted using a one-way ANOVA Tukey test shown with letters, and one-tailed Student’s *t*-tests were calculated for FAs and their controls, with *P*-value significance indicated on the left of the graphs.

Total counts are shown in Fig. 3, with results from three coded experiments, alongside the percentage of pink nodules. In MFA and VFA treatments, mean total nodule number was only slightly increased (not significantly), but the number of PNs at 21 d was significantly increased compared with all other treatments. The pink colour of large PNs is indicative that *Rhizobium* are actively fixing N within the nodule, caused by the presence of leghaemoglobin (A. Liu *et al.*, 2018). Therefore, FA treatment may affect the rate of N fixation and thus increase plant vegetative growth. In addition, testing of *M. sativa* on sterile agar plates with FA application alongside control treatments showed that the significant vegetative growth increase was found only when plates were inoculated with the *S. meliloti* strain. Plate experiment phenotypes of vegetative biomass, nodule number, and root biomass of cv. Daisy showed that increases of the former two are specific to *Rhizobium* inoculation (Supplementary Fig. S6).

It is possible that FAs may also directly influence N-fixing bacteria such as *Sinorhizobium*. This has been reported in other studies (Little *et al.*, 2014; Xu *et al.*, 2018); however, these studies did not include nutritional controls. Improved symbiotic association of leguminous crops with *Rhizobium* is important with the current emphasis on growing more leguminous crops globally, due to their N-fixing activity (Lüscher *et al.*, 2014; Preissel *et al.*, 2015; Foyer *et al.*, 2016; Iannetta *et al.*, 2016; Reckling *et al.*, 2016). For example, the fixing of atmospheric N_2_ in legume/grass pastures has been estimated as 13–682 kg N ha^–1^ year^–1^ (Ledgard and Steele, 1992). *Medicago sativa* itself has been estimated to fix up to 350 kg N ha^–1^ year^–1^, providing an N fixation rate of 0.021×vegetative dry matter+16.9 (*R*^2^=0.91) (Carlsson and Huss-Danell, 2003), regardless of soil status or geographic location. Increased vegetative growth in *M. sativa* due to improved symbiosis with *Rhizobium* could have implications for the yields of other cultivated legumes.

### Microbial growth is affected by fulvic acid

**Fig. 4.**
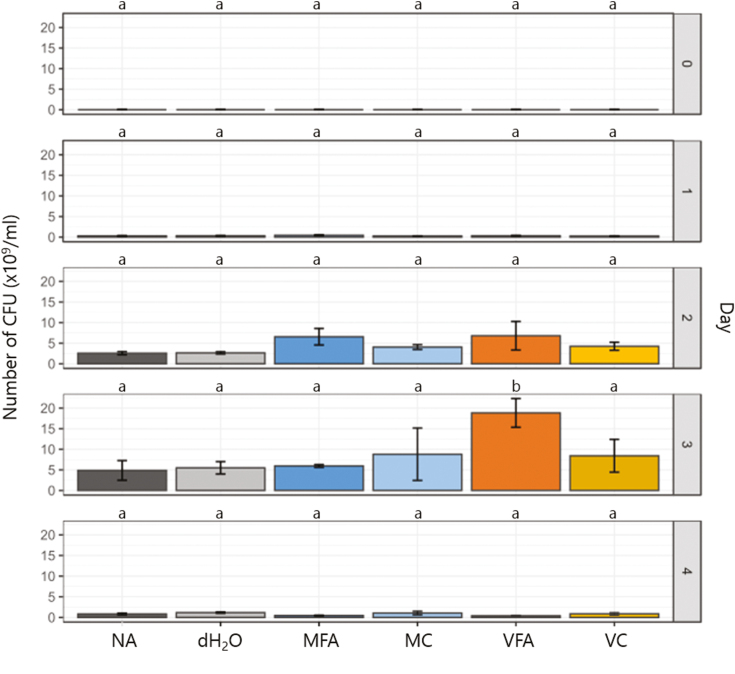
Effects of fulvic acid on the growth of *Sinorhizobium meliloti* in culture, compared with controls. TY cultures containing treatments as follows were inoculated with *S. meliloti*: NA in dark grey; dH_2_O in grey; 0.5% MFA in blue; 0.5% MC in light blue; 1% VFA in orange; 1% VC in yellow. Average colony-forming unit (CFU) counts were obtained from triplicate samples on days 0–4 of incubation with shaking at 220 rpm at 28 °C. Average counts for three separate experiments (three individual experimental replicates (on separate days), each with three technical replicates) were calculated and are shown above with the SD. Multiple comparisons between treatments were conducted using a one-way ANOVA Tukey test shown with letters.

To determine if FAs affect growth of *S. meliloti* in the absence of plants, microbial growth in liquid culture was tested. Cultures of FAs or controls inoculated with *S. meliloti* were grown over 4 d with cell density tested using the standard microbial technique of CFU counts. The mean cell density results of three independent experiments are shown in Fig. 4. No effect of FAs on cell density was found at 0–1 d. At 2 d, both FA-treated cultures had a higher cell density than their nutritional controls; MFA measured 6.56×10^9^ compared with MC at 4.07×10^9^; VFA measured 6.81×10^9^ compared with VC at 4.26×10^9^. By 3 d, the MFA-treated culture did not differ in its cell density from any controls. Conversely, VFA had a significantly higher cell density of 1.88×10^10^. These results indicate that adding FAs to liquid cultures can increase growth of *S. meliloti*, with a similar result demonstrated in a study comparing the presence of a HS substance with no addition (Xu *et al.*, 2018). Furthermore, the effect of FAs on increasing microbial cell growth without the presence of a plant interaction agrees with other published studies; HS addition has been shown to increase growth of *Bradyrhizobium liaoningense* in liquid culture (Guo Gao *et al.*, 2015) and increase general microbial population growth in soil microbial cells (Visser, 1985). In contrast, a study of *Candida utilis* found no growth change with HS application, so the response may be specific to certain taxa (McLoughlin and Küster, 1972). It is possible that FA is able to affect the growth of other soil microbial populations which may also increase plant vegetative yields. This may include other important *Rhizobium* species for *Medicago* relatives, but, moreover, species of *Streptomyces*, *Bacillus*, and arbuscular mycorrhiza fungi (Schirawski and Perlin, 2018). Comparing the nutritional controls (MC and VC) with distilled water treatments (dH_2_O) showed that there was no significant nutritional component to the effect of FA application (see Fig. 4).

### RNA-seq demonstrates high levels of transcriptional changes in roots following FA treatment after 3 d

Transcriptional changes were investigated using RNA-seq for *M. sativa* shoot and root tissues treated with either VFA or its nutritional control VC, on the day of treatment (day 0) or 3 d after the treatment (day 3). DE transcripts were analysed with *de novo* transcriptome assembly, performed to negate for initial bias of other reference alignment such as *M. truncatula* in subsequent analysis. *De novo* transcriptome assembly was successful for building a scaffold, shown in Supplementary Table S7, with similar alignment rates of all transcripts for available *M. truncatula* references (data not shown).

**Fig. 5.**
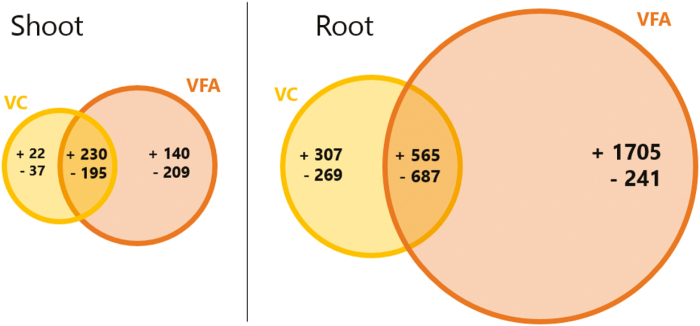
Differentially expressed transcripts in *Medicago sativa* shoot and root tissue with treatments of either VFA (orange) or VC (yellow). RNA-seq was carried out on whole shoot and root RNA samples taken on the day of treatment (day 0) or 3 d after treatment (day 3). Transcripts from *de novo* transcriptome assembly with both an absolute log fold change of 0.585 (1.5×FC) and a false discovery rate- (FDR) adjusted *P*-value (*q*-value) <0.05 were considered as differentially expressed (DE); DE transcripts significantly expressed between treatments at day 0 were removed to negate for false positives due to experimental variance. The Venn diagram shows up-regulated (+) and down-regulated (–) DE transcripts for both treatments between day 0 and day 3, including those which are shared (overlapping region). This difference in DE transcript number is emphasized by differing sizes of the circles in the plot.

DE transcripts for VFA and VC between the two time points were investigated, with transcripts requiring both an absolute log FC of 0.585 (1.5×FC) and an FDR-adjusted *P*-value (*q*-value) <0.05; Supplementary Fig. S7 shows those between day 0 and day 3 for VFA application in root samples. Figure 5 shows the number of up-regulated (+) and down-regulated (–) DE transcripts in shoots and roots *of M. sativa* following VFA and/or VC treatment. This result reveals that most DE transcripts (1705 up-regulated and 241 down-regulated) for VFA treatment occurred in the root tissue. This is compared with 140 up-regulated and 209 down-regulated DE transcripts in the shoot. This study shows that FA as VFA can induce substantial transcriptional changes in *M. sativa* after only 3 d, with the root showing far higher numbers of DE transcripts than shoots.

Further analysis of DE transcripts can be found in the Supplementary data, with descriptions of BLAST results and analysis of both tissue types, and functional annotation of GO terms and enrichment testing of root samples only. Most DE isoforms had homologues in closely related legume species (Supplementary Fig. S8a). Many successful BLAST hits were recorded for each transcript sequence (Supplementary Fig. S8b), with significant hits in most having an extremely low E-value close to zero (Supplementary Fig. S8c). Following annotation, the GO terms in Supplementary Table S9 and Supplementary Fig. S9 demonstrated that root FA-induced transcriptional changes are wide ranging for biological process. There are high GO term hits for processes affected by VFA treatment including those regulating transcription and translation, and those associated with oxidation–reduction, metabolism, and transport. The GO analysis provided evidence that VFA very quickly affects crucial pathways in both C and N metabolism, as well as cell wall modification. This rapid transcriptional effect is likely to induce the later yield effect in vegetative tissue. There was an indication of changes in responses to defence, stress, and bacteria. These may be linked to response to symbiotic bacteria such as *S. meliloti*; at this developmental stage, nodulation can begin to be established, and it is well documented that important nodulation genes and factors are associated with defence responses through their evolution and function (Kouchi *et al.*, 2004; Lohar *et al.*, 2006; Libault *et al.*, 2010; Chen *et al.*, 2017; Clúa *et al.*, 2018).

Enrichment testing shown in Supplementary Table S10 further provides evidence that VFA particularly up-regulated biological processes associated with N metabolism, similar to findings in Supplementary Fig. S9. Changes in N metabolism in the roots of legume species are associated with increases in nodulation signalling during initiation of symbiosis (A. Liu *et al.*, 2018). The quick response in transcription in the roots suggests why there is a larger biomass increase after VFA treatment, probably through stimulated N supply to the legume via nodules or uptake by the roots. Responses to bacteria were enriched, providing further evidence from GO analysis that an effect on nodulation initiation may be the cause of such a change. Moreover, other important processes required for new root development and nodule growth showed enrichment, including cell wall biogenesis and organization. Molecular function testing showed enrichment in root nutrient transporter activity following VFA treatment, as well as enrichment of serine hydrolase activity, which has wide-ranging catalytic activity in plants (Kaschani *et al.*, 2012; Mindrebo *et al.*, 2016).

### Transcriptome analysis shows preferential enrichment of nodulation regulation and signalling-related genes

The genes identified from the above analysis in the root which were significantly induced following VFA treatment were noted to overlap considerably with those in studies of early initiation of nodulation in legumes (El Yahyaoui *et al.*, 2004; Kouchi *et al.*, 2004; Moreau *et al.*, 2011; Hayashi *et al.*, 2012; O’Rourke *et al.*, 2013; Alves-Carvalho *et al.*, 2015; Larrainzar *et al.*, 2015; Kant *et al.*, 2016). To further investigate this, DE transcripts were compared with those which have been categorized as specific early symbiotic root nodulation genes in *M. truncatula* by Roux *et al.* (2014). In that study, laser-capture microdissection of roots and nodules was coupled with RNA-seq (Roux *et al.*, 2014). This provided a robust list of genes induced at various stages of nodulation especially in early initiation. Table 1 details those DE transcripts up-regulated in the root following VFA treatment which are early genes required for the signalling and regulation of nodulation; annotations are available for many of these. These included an array of transcription factors and domains including *Myb/SANT-like DNA-binding domain protein*, *AP2-like ethylene-responsive transcription factor*, and *zinc finger MYM-type protein 1-like*; AP2/ERF transcription factors are known to control nodule number and differentiation (Middleton *et al.*, 2007; Vernie et al., 2008;Wang *et al.*, 2010; Peng *et al.*, 2017). Many leucine-rich repeat receptor-like kinases (LRR RLKs) and other receptor kinases were found to be highly enriched, for example *LysM domain receptor-like kinase*; many LRR RLKs including *CLAVATA* protein homologues signal root development and nodulation induction (Krusell *et al.*, 2002; Schnabel *et al.*, 2005; Oka-Kira and Kawaguchi, 2006; Mortier *et al.*, 2010; Lim *et al.*, 2011; Reid *et al.*, 2011), and *LysM-type receptor-like kinases* perceive early *Rhizobium* signals (Amor *et al.*, 2003; Limpens *et al.*, 2003; Madsen *et al.*, 2003; Radutoiu *et al.*, 2003; Knogge and Scheel, 2006; Indrasumunar *et al.*, 2011; Popp and Ott, 2011; Kawaharada *et al.*, 2015; Zipfel and Oldroyd, 2017).

**Table 1.** Enriched DE transcripts in VFA roots which are putatively classed as highly preferential nodulation regulatory genes and nodule-associated signalling-related genes as in Roux *et al.* (2014)

Gene/protein ID	Description	Annotation	logFC	*q*-value
XP_024635034	*Myb/SANT-like DNA-binding domain protein*	TF MYB	5.35	1.90E^-06^
PNX91228	*Putative CC-NBS-LRR resistance protein*	LRR	4.98	1.23E^-11^
ABD33274, AES59362, RHN77255	*RALF-like protein*	Calcium/lipid binding	4.74	4.63E^-06^
RIA81513	*Calnexin*	Calcium/lipid binding	4.67	6.36E^-09^
RHN49201	*Wall-associated receptor kinase-like 20*	RLK	4.60	3.35E^-05^
KEH36571, RHN72042	*CLAVATA3/ESR (CLE)-related protein*	Ser/Thr protein kinase	4.36	2.44E^-07^
KEH28705, RHN58556	*Putative LRR-domain, L domain-containing protein*	LRR	4.29	6.43E^-05^
XP_003612592, AES95550, RHN54652	*RING-H2 finger protein ATL52-like*	TF ZnFg C2H2	4.28	1.63^–75^
XP_024641562	*AP2-like ethylene-responsive transcription factor*	TF AP2/ERF	4.08	1.74^-04^
XP_003594815, AES65066, RHN73104	*COBRA-like protein 7*	COBRA	4.00	1.78E^-07^
XP_003598348, AES68599, RHN65475	*F-box protein interaction domain protein*	F‐box protein	3.75	4.90E^-07^
AES76072, AES76110, RHN52304	*NDR1/HIN1-like protein 10*	NHL	3.67	2.52E^-04^
RHN60433	*Disease resistance protein (TIR-NBS-LRR class)*	LRR RLK	3.64	7.66E^-06^
XP_013443270, KEH17295, RHN51739	*Cytokinin hydroxylase-like*	CK activated	3.60	6.29E^-06^
XP_013466350, KEH40391, RHN77806	*Receptor-like protein kinase*	RLK	3.55	4.72E^-06^
XP_003604023, AES74274	*COBRA-like protein 1*	COBRA	3.53	7.35E^-07^
RGB31681	*Calcium-binding protein*	Calcium/lipid binding	3.49	1.84E^-06^
RHN72504	*Probable inactive receptor kinase At2g26730*	RLK	3.42	1.32E^-05^
XP_003613167, AES96125, RHN55010	*l* *-tryptophan-pyruvate aminotransferase 1*	TAA1‐like	3.39	2.54E^-04^
AES69839	*LRR-P-loop containing nucleoside triphosphate hydrolase*	LRR	3.32	7.54E^-06^
AES91737	*F-box/kelch-repeat protein*	F-box protein	3.32	6.92E^-06^
XP_024637477	*Disease resistance protein (TIR-NBS-LRR class)*	LRR	3.24	1.65E^-05^
EXX59026	*WD40 repeat-like protein*	TF WD	3.22	1.01E^-04^
XP_024631685, RHN72543	*Mitogen-activated protein kinase kinase kinase 18-like*	STY	3.14	2.55E^-05^
ABD28520	*Protein RRP6-like 2*	CK activated	3.09	9.33E^-05^
XP_013451548, KEH25576, RHN50766	*Ankyrin repeat/protein kinase domain-containing protein 1*	TF ERF	3.06	2.97E^-05^
AES95938	*Disease resistance protein (TIR-NBS-LRR class), putative*	LRR RLK	3.01	1.91E^-05^
RZB96753	*Probable LRR receptor-like Ser/Thr-protein kinase*	LRR RLK	2.99	5.78E^-05^
KHN26259	*Zinc finger MYM-type protein 1-like*	TF Zn finger	2.95	1.31E^-04^
XP_013451184, KEH25223, RHN50327	*Protein NSP-interacting kinase 1*	NSP	2.94	3.50E^-05^
RHN42361	*Kinase RLK-Pelle-WAK-LRK10L-1 family*	RLK	2.89	1.29E^-04^
RIA84146	*Ca* ^*2+*^ *:H* ^*+*^ *antiporter*	Calcium/lipid binding	2.87	8.02E^-05^
AES60803	*F-box plant-like protein*	F-box protein	2.78	1.71E^-04^
XP_013457946, KEH31977, RHN63702	*Putative LRR-containing protein*	LRR RLK	2.78	1.60E^-04^
RIA97789	*ARM repeat-containing protein*	E3 ligase	2.72	2.11E^-04^
XP_013445632	*G-type lectin S-receptor-like Ser/Thr-protein kinase*	Ser/Thr protein kinase	2.71	1.84E^-04^
AES73438	*Plant regulator RWP-RK*	NLP	2.70	1.87E^-04^
KEH38435	*Rpp4C3*	CK activated	2.69	1.22E^-04^
RIA81779	*YIF1-domain-containing protein*	TF AP2/ERF	2.69	1.26E^-04^
AES61923, RHN81250	*C3HC4-type RING zinc finger protein*	TF Zn finger	2.68	1.90E^-04^
XP_024633471.1	*LysM domain receptor-like kinase 3*	LysM receptor kinase	2.68	1.41E^-04^
XP_024625794	*Putative receptor-like protein kinase*	RLK	2.66	4.17E^-05^
RHN81081	*Proline-rich protein 1-like*	PRP	2.63	5.03E^-05^
PF04909	*Nodulin-6*	NIP	2.56	2.00E^-04^
XP_003615114, AES98072, RHN56135	*Nodulin-26*	NIP	2.56	6.80E^-05^
XP_013450575, RHN49450	*L-type lectin-domain containing receptor kinase IX.1-like*	RLK	2.49	5.82E^-05^
XP_013462891, KEH36925, RHN72571	*Chitin elicitor receptor kinase 1-like*	LysM receptor kinase	2.48	9.25E^-06^
XP_003601076.1, AES71327	*Nodulation-signalling pathway 2 protein*	NSP	2.11	1.43E^-05^
XP_024641514, AES76606, RHN52721	*Putative NF-X1-type zinc finger protein NFXL1-like protein*	NFX1-type zinc finger	1.75	1.68E^-05^
XP_013460228, KEH34259, RHN67624	*Non-specific phospholipase*	Phospholipase A2	1.52	4.71E^-06^
XP_024625319	*U-box domain-containing protein 33 isoform X1*	MtPUB	1.37	1.14E^-04^
XP_003631134, AET05610, RHN43936	*Probable inactive receptor kinase At1g48480*	Kinase	1.15	1.75E^-04^
XP_003616008, AES98966, RHN56723	*CBL-interacting serine/threonine-protein kinase 11*	Calcium binding, Ser/Thr protein kinase	1.04	1.40E^-04^
RHN48771	*NDR1/HIN1-like protein 1*	NHL	0.88	3.71E^-05^

The table includes a description of the protein, available gene/protein IDs, the annotation type, log fold change (logFC), and *q*-value for each DE transcript.

Genes required in bacteria- and hormone-induced plant responses were found to be up-regulated, for example *NDR1/HIN1-like protein 10*, *protein RRP6-like 2*, and *cytokinin hydroxylase-like transcripts*; an increase of *Pathogenesis-related proteins* can be induced in early symbiotic infection, before adequate *Rhizobium* suppression, rather than being in relation to a pathogen response (Kouchi *et al.*, 2004; Lohar *et al.*, 2006; Libault *et al.*, 2010; Nakagawa *et al.*, 2011; Popp and Ott, 2011; Oldroyd, 2013; Clúa *et al.*, 2018). Important chitin regulatory genes are also detected to be changed in their expression by VFA treatment. This may affect lipochitooligosaccharide recognition as the key signal in initiating legume–*Rhizobium* symbiosis (Dénarié *et al.*, 1996; Reddy *et al.*, 1998; Liang *et al.*, 2014; Muñoz *et al.*, 2014; Bozsoki *et al.*, 2017).

Finally, many nodulation-specific genes were enriched such as *nodulation-signaling pathway (NSP) proteins*, *NSP-interacting kinases*, and *nodulins*; *nodulin* is crucial in early nodule development (Legocki and Verma, 1980; Scheres *et al.*, 1990; van de Wiel *et al.*, 1990; Kouchi and Hata, 1993; Rivers *et al.*, 1997; Gamas *et al.*, 1998; Mathis *et al.*, 1999; Becker *et al.*, 2001; Marsh *et al.*, 2007; Kant *et al.*, 2016; Roberts and Routray, 2017; Y.-C. Liu *et al.*, 2018) including in *M. sativa* (Fang and Hirsch, 1998; Cheng *et al.*, 2000; Pringle and Dickstein, 2004; Lafuente *et al.*, 2010).

The increase in transcription of these genes upon VFA treatment is a strong indication that this HS is associated with inducing early nodulation in *M. sativa*. The effect could be by influencing the plant itself in its response to symbiosis, for example a priming effect of VFA for subsequently inducing infection by the symbiont (Berg *et al.*, 1989; Harris *et al.*, 2005; Alimadadi *et al.*, 2010). FA may be able to change the C:N metabolic balance of the plant and thus impact on the regulatory mechanisms of promoting symbiotic nodulation processes (Libault, 2014), or the effect could be a consequence of the treatment on the symbiont causing a nodule number increase. VFA may contain a specific nutritional aid, not adequately controlled for in VC application, which boosts symbiotic *Sinorhizobium* growth in soil and thus makes nodulation happen more rapidly (Singleton and Tavares, 1986; Thies *et al.*, 1991). Similarly, VFA may decrease the inhibitory role of N in soil on nodulation and thus also encourage nodulation to occur with the symbiont and plant (Beauchamp *et al.*, 2001; Zeijl *et al.*, 2018, preprint). This is unlikely to be due to the low N content of the soil used in testing, but should be considered.

### Vegetative growth effect was recorded in independent field trials

**Fig. 6.**
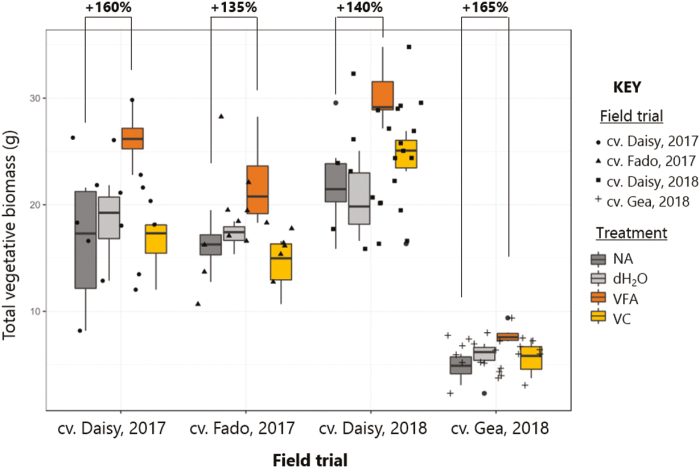
Vegetative biomass of *Medicago sativa* cultivars in independent field trials following treatment with a fulvic acid or controls. Treatments were applied to field plots at the beginning of establishment, and vegetative yields were assessed before the first cut of the growing season; an area of 625 cm^2^ was sampled and total vegetative tissue was dried for biomass (in g). Treatments were: no addition (NA in dark grey); deionized water (dH_2_O in grey); 1% VFA (VFA in orange); and 1% VC (VC in yellow). Three trials of four cultivars were run over 2 years. In 2017, trials were performed at Dengie Crops Ltd (Southminster, Essex) with four plots per treatment of both cvs Daisy and Fado. In 2018, the trials were at both Blankney Estates Ltd (Blankney, Lincolnshire) and A Poucher and Sons (Bardney Dairies) Ltd (Market Rasan, Lincolnshire) with six plots per treatment of cvs Daisy and Gea, respectively. Individual plot samples are shown in black data points as indicated, and boxplots are for individual cultivar trials. Relative average increase in yield of VFA-treated plots compared with NA is shown as a percentage above the graph to the nearest 5%.

Figure 6 shows a comparison of the vegetative biomass of *M. sativa* cultivars in independent field trials following treatment with an FA or controls, in order to check if the FA treatment effect on *M. sativa* in glasshouse experiments could be demonstrated in the field. Over 2 years, four trials were conducted at three dedicated forage crop cultivation farms. Trial plots treatments were one of NA, dH_2_O, VFA, or VC at early establishment of *M. sativa* (April–June). These plots were grown in accordance with site standard management practices for UK forage crop cultivation. Prior to the first harvest of the season (May–July), vegetative biomass was recorded for a sample from each treatment plot, shown in Fig. 6 for each experiment. Although different cultivars were tested at the various sites, for each experiment VFA-treated plots had increased vegetative biomass. This increased growth compared with NA or VC was 135–165%, which is only slightly lower than measurements from glasshouse experiments of 167–185%. The biomass increased for all vegetative tissues, both shoot and leaf.

The nutritional content of *M. sativa* tissue from each treatment plot was also assessed for the 2018 trials. The results of one 2018 trial are shown in Supplementary Fig. S11 with samples of total vegetative biomass measurements, total chlorophyll, and total protein of samples. Although there was a significant difference in vegetative biomass, no significant difference in nutritional content was shown across any treatment. The other 2018 trial had similar results, with NA, dH_2_O, VFA, and VC plots having an average chlorophyll and total protein content as follows; 2.91% and 17.87%; 2.61% and 17.96%; 2.83% and 18.21%; and 2.63% and 17.97%. These measurements show that the yield effect of VFA treatment on *M. sativa* is not linked to changes in tissue nutritional content. The enhanced vegetative growth from nodule stimulation did not result in increased protein storage. It is possible that FA and other HS treatments in other studies may replicate C-containing exudates usually released by plants to aid in symbiosis initiation, which in turn stimulates activity of *Rhizobium*. This increases nodulation signalling which encourages symbiosis, and results in higher nodule activity, and thus yield is increased.

It has been suggested that HS have a crucial active ingredient or ‘hormone’, such as an auxin-like molecule (Nardi et al., 1994, 2002; Russell *et al.*, 2006; Canellas *et al.*, 2010; Trevisan et al., 2010*a*, *b*). However, based on the analysis in this study, no such auxin-like molecule was detected, and both commercial applications were found to be different from one another. It is possible that past studies lacking nutritional controls may have given auxin-like growth stimulation, as the plants may have been subjected to suboptimal nutritional supply before treatment and growth effects after application could be interpreted as due to a hormonal stimulus. Although FA did promote growth in legumes. the response may be complicated by the mixture of many compounds in the product. By performing a chemical fractionation of FAs, it may be possible to find several common components with synergistic effects on nodulation.

In conclusion, we have demonstrated a specific stimulatory effect of FA treatment on the early stages of nodulation in *M. sativa* in both the glasshouse and the field. The FA treatment significantly enhanced biomass production and may be relevant for other legume crops.

## Supplementary data

Supplementary data are available at *JXB* online.

**Table S1.** MFA detectable content in mg l^–1^ for individual elements by ICP-OES and ICP-MS for a 0.5% solution.

**Table S2.** VFA detectable content in mg l^–1^ for individual elements by ICP-OES and ICP-MS for a 0.5% solution.

**Table S3.** MFA control solution (MC) components for a 0.5% application.

**Table S4.** VFA control solution (VC) components for a 1.0% application.

**Table S5**. Initial RNA quality check for RNA-seq samples.

**Table S6**. Sequencing quality check for RNA-seq samples.

**Table S7.** Pseudoalignment summary of RNA-seq samples with *de novo* transcriptome assembly.

**Table S8.** Differentially expressed (DE) transcript list for 0VFA versus 3VFA.

**Table S9**. Functional annotation of root DE transcripts where P=Biological Process, F=Molecular Function, and C=Cellular Compartment.

**Table S10**. Enrichment testing of root DE transcripts.

**Table S11**. Quantitative reverse transcription–PCR (qRT–PCR) of a subset of root DE transcripts to confirm RNA-seq.

**Fig. S1.** Gas chromatogram spectra of (a) citric acid standard and (b) MFA, with (c and d) NIST Atomic Spectra database 1A v14 matches for citric acid, 4TMS derivative, and trimethyl TMS derivative.

**Fig. S2.** Gas chromatogram spectra of (a) PEG-400 standard and (b) VFA, with (c) NIST Atomic Spectra database 1A v14 matches for poly(ethylene glycol) (heptaethylene glycol).

**Fig. S3.** MFA ^13^C-NMR analysis for carbon compounds in the evaporated sample.

**Fig. S4.** VFA ^13^C-NMR analysis for carbon compounds in the evaporated sample.

**Fig. S5.** Vegetative biomass of forage crops following one of two fulvic acid treatments relative to dH_2_O.

**Fig. S6.** Vegetative and nodule phenotypes of plate-grown *Medicago sativa* cv. Daisy following treatment with fulvic acids or controls, with or without inoculation of *Sinorhizobium meliloti*.

**Fig. S7.** Volcano plot of DE transcripts as log fold change (logFC) between 0 day and 3 day for VFA treatment RNA samples.

**Fig. S8.** BLAST sequencing results for DE transcripts of VFA treatment from *de novo* RNA-seq analysis, showing results for root tissue only.

**Fig. S9.** ‘Biological Process’ GO term hits for individual DE transcripts from VFA treatment of *Medicago sativa* roots.

**Fig. S10.** Field plots for fulvic acid trials performed in 2017 and 2018.

**Fig. S11.** Vegetative tissue of *Medicago sativa* first cut following fulvic acid treatment compared with a control, from field trial plots.

eraa283_suppl_Supplementary_Figures_and_Tables

eraa283_suppl_Supplementary_Tables

## References

[CIT0001] Alimadadi A, Jahansouz MR, Besharati H, Tavakol Afshari R 2010 Evaluating the effects of phosphate solubilizing microorganisms, mycorrhizal fungi and seed priming on nodulation of chickpea. Iranian Journal of Soil Research 24, 44–53.

[CIT0002] Altschul SF, Gish W, Miller W, Myers EW, Lipman DJ 1990 Basic local alignment search tool. Journal of Molecular Biology 215, 403–410. 2231712 10.1016/S0022-2836(05)80360-2

[CIT0003] Altschul SF, Madden TL, Schäffer AA, Zhang J, Zhang Z, Miller W, Lipman DJ 1997 Gapped BLAST and PSI-BLAST: a new generation of protein database search programs. Nucleic Acids Research 25, 3389–3402. 9254694 10.1093/nar/25.17.3389 PMC146917

[CIT0004] Alves-Carvalho S, Aubert G, Carrère S, et al. 2015 Full-length de novo assembly of RNA-seq data in pea (*Pisum sativum* L.) provides a gene expression atlas and gives insights into root nodulation in this species. The Plant Journal 84, 1–19. 26296678 10.1111/tpj.12967

[CIT0005] Amor BB, Shaw SL, Oldroyd GE, Maillet F, Penmetsa RV, Cook D, Long SR, Dénarié J, Gough C 2003 The NFP locus of *Medicago truncatula* controls an early step of Nod factor signal transduction upstream of a rapid calcium flux and root hair deformation. The Plant Journal 34, 495–506. 12753588 10.1046/j.1365-313x.2003.01743.x

[CIT0006] Ashburner M, Ball CA, Blake JA, et al. 2000 Gene ontology: tool for the unification of biology. The Gene Ontology Consortium. Nature Genetics 25, 25–29. 10802651 10.1038/75556 PMC3037419

[CIT0007] Aydin A, Kant C, Turan M 2012 Humic acid application alleviate salinity stress of bean (*Phaseolus vulgaris* L.) plants decreasing membrane leakage. African Journal of Agricultural Research 7, 1073–1086.

[CIT0008] Beauchamp CJ, Kloepper JW, Shaw JJ, Chalifour FP 2001 Root colonization of faba bean (*Vicia faba* L.) and pea (*Pisum sativum* L.) by *Rhizobium leguminosarum* bv. *viciae* in the presence of nitrate-nitrogen. Canadian Journal of Microbiology 47, 1068–1074. 11822831 10.1139/w01-113

[CIT0009] Becker JD, Moreira LM, Kapp D, Frosch SC, Pühler A, Perlic AM 2001 The nodulin *VfENOD18* is an ATP-binding protein in infected cells of *Vicia faba* L. nodules. Plant Molecular Biology 47, 749–759. 11785936 10.1023/a:1013664311052

[CIT0010] Benson DA, Cavanaugh M, Clark K, Karsch-Mizrachi I, Lipman DJ, Ostell J, Sayers EW 2013 GenBank. Nucleic Acids Research 41, D36–D42. 23193287 10.1093/nar/gks1195 PMC3531190

[CIT0011] Benson DA, Karsch-Mizrachi I, Lipman DJ, Ostell J, Wheeler DL 2005 GenBank. Nucleic Acids Research 33, D34–D38. 15608212 10.1093/nar/gki063 PMC540017

[CIT0012] Berg RK, Jawson MD, Franzluebbers AJ, Kubik KK 1989 *Bradyrhizobium japonicum* inoculation and seed priming for fluid-drilled soybean. Soil Science Society of America 53, 1712–1717.

[CIT0013] Bezuglova OS, Polienko EA, Gorovtsov AV, Lyhman VA, Pavlov PD 2017 The effect of humic substances on winter wheat yield and fertility of ordinary chernozem. Annals of Agrarian Science 15, 239–242.

[CIT0014] Binns D, Dimmer E, Huntley R, Barrell D, O’Donovan C, Apweiler R 2009 QuickGO: a web-based tool for Gene Ontology searching. Bioinformatics 25, 3045–3046. 19744993 10.1093/bioinformatics/btp536 PMC2773257

[CIT0015] Bozsoki Z, Cheng J, Feng F, Gysel K, Vinther M, Andersen KR, Oldroyd G, Blaise M, Radutoiu S, Stougaard J 2017 Receptor-mediated chitin perception in legume roots is functionally separable from Nod factor perception. Proceedings of the National Academy of Sciences, USA 114, E8118–E8127. 10.1073/pnas.1706795114 PMC5617283 28874587

[CIT0016] Bray NL, Pimentel H, Melsted P, Pachter L 2016 Near-optimal probabilistic RNA-seq quantification. Nature Biotechnology 34, 525–527. 10.1038/nbt.3519 27043002

[CIT0017] Calvo P, Nelson L, Kloepper JW 2014 Agricultural uses of plant biostimulants. Plant and Soil 383, 3–41.

[CIT0018] Camacho C, Coulouris G, Avagyan V, Ma N, Papadopoulos J, Bealer K, Madden TL 2009 BLAST+: architecture and applications. BMC Bioinformatics 10, 421. 20003500 10.1186/1471-2105-10-421 PMC2803857

[CIT0019] Canellas LP, Olivares FL 2014 Physiological responses to humic substances as plant growth promoter. Chemical and Biological Technologies in Agriculture 1, 3.

[CIT0020] Canellas LP, Piccolo A, Dobbss LB, Spaccini R, Olivares FL, Zandonadi DB, Façanha AR 2010 Chemical composition and bioactivity properties of size-fractions separated from a vermicompost humic acid. Chemosphere 78, 457–466. 19910019 10.1016/j.chemosphere.2009.10.018

[CIT0021] Carbon S, Ireland A, Mungall CJ, Shu S, Marshall B, Lewis S; AmiGO Hub; Web Presence Working Group 2009 AmiGO: online access to ontology and annotation data. Bioinformatics 25, 288–289. 19033274 10.1093/bioinformatics/btn615 PMC2639003

[CIT0022] Carlsson G, Huss-Danell K 2003 Nitrogen fixation in perennial forage legumes in the field. Plant and Soil 253, 353–372.

[CIT0023] Chang Z, Liu Y, Dong H, Teng K, Han L, Zhang X 2016 Effects of cytokinin and nitrogen on drought tolerance of creeping bentgrass. PLoS One 11, e0154005. 27099963 10.1371/journal.pone.0154005 PMC4839601

[CIT0024] Chen T, Duan L, Zhou B, Yu H, Zhu H, Cao Y, Zhang Z 2017 Interplay of pathogen-induced defense responses and symbiotic establishment in *Medicago truncatula*. Frontiers in Microbiology 8, 973. 28611764 10.3389/fmicb.2017.00973 PMC5447765

[CIT0025] Cheng X-G, Nomura M, Takane K, Kouchi H, Tajima S 2000 Expression of two uricase (*Nodulin-35*) genes in a non-ureide type legume, *Medicago sativa*. Plant & Cell Physiology 41, 104–109. 10750714 10.1093/pcp/41.1.104

[CIT0026] Clúa J, Roda C, Zanetti EM, Blanco AF 2018 Compatibility between legumes and *Rhizobia* for the establishment of a successful nitrogen-fixing symbiosis. Genes 9, 125. 10.3390/genes9030125 PMC5867846 29495432

[CIT0027] Conesa A, Götz S, García-Gómez JM, Terol J, Talón M, Robles M 2005 Blast2GO: a universal tool for annotation, visualization and analysis in functional genomics research. Bioinformatics 21, 3674–3676. 16081474 10.1093/bioinformatics/bti610

[CIT0028] Conesa A, Madrigal P, Tarazona S, et al. 2016 A survey of best practices for RNA-seq data analysis. Genome Biology 17, 13. 26813401 10.1186/s13059-016-0881-8 PMC4728800

[CIT0029] Costa-Silva J, Domingues D, Lopes FM 2017 RNA-Seq differential expression analysis: an extended review and a software tool. PLoS One 12, e0190152. 29267363 10.1371/journal.pone.0190152 PMC5739479

[CIT0030] Daneshvar Hakimi Maibodi N, Kafi M, Nikbakht A, Rejali F 2015 Effect of foliar applications of humic acid on growth, visual quality, nutrients content and root parameters of perennial ryegrass (*Lolium perenne* L.). Journal of Plant Nutrition 38, 224–236.

[CIT0031] Dénarié J, Debellé F, Promé JC 1996 *Rhizobium* lipo-chitooligosaccharide nodulation factors: signaling molecules mediating recognition and morphogenesis. Annual Review of Biochemistry 65, 503–535. 10.1146/annurev.bi.65.070196.002443 8811188

[CIT0032] Dobbss LB, Medici LO, Peres LEP, Pino-Nunes LE, Rumjanek VM, Façanha AR, Canellas LP 2007 Changes in root development of *Arabidopsis* promoted by organic matter from oxisols. Annals of Applied Biology 151, 199–211.

[CIT0033] El Yahyaoui F, Küster H, Ben Amor B, et al. 2004 Expression profiling in *Medicago truncatula* identifies more than 750 genes differentially expressed during nodulation, including many potential regulators of the symbiotic program. Plant Physiology 136, 3159–3176. 15466239 10.1104/pp.104.043612 PMC523376

[CIT0034] Fang Y, Hirsch AM 1998 Studying early nodulin gene *ENOD40* expression and induction by nodulation factor and cytokinin in transgenic alfalfa. Plant Physiology 116, 53–68. 9449836 10.1104/pp.116.1.53 PMC35188

[CIT0035] FAO, IFIF 2010 Good practices for the feed industry—implementing the codex alimentarius code of practice on good animal feeding. Rome: FAO/IFIF.

[CIT0036] Foyer CH, Lam HM, Nguyen HT, et al. 2016 Neglecting legumes has compromised human health and sustainable food production. Nature Plants 2, 16112. 28221372 10.1038/nplants.2016.112

[CIT0037] Gamas P, de Billy F, Truchet G 1998 Symbiosis-specific expression of two *Medicago truncatula* nodulin genes, *MtN1* and *MtN13*, encoding products homologous to plant defense proteins. Molecular Plant-Microbe Interactions 11, 393–403. 9574507 10.1094/MPMI.1998.11.5.393

[CIT0038] García AC, Santos LA, Izquierdo FG, Sperandio MVL, Castro RN, Berbara RLL 2012 Vermicompost humic acids as an ecological pathway to protect rice plant against oxidative stress. Ecological Engineering 47, 203–208.

[CIT0039] Götz S, García-Gómez JM, Terol J, Williams TD, Nagaraj SH, Nueda MJ, Robles M, Talón M, Dopazo J, Conesa A 2008 High-throughput functional annotation and data mining with the Blast2GO suite. Nucleic Acids Research 36, 3420–3435. 18445632 10.1093/nar/gkn176 PMC2425479

[CIT0040] Grabherr MG, Haas BJ, Yassour M, et al. 2011 Full-length transcriptome assembly from RNA-Seq data without a reference genome. Nature Biotechnology 29, 644–652. 10.1038/nbt.1883 PMC3571712 21572440

[CIT0041] Guijas C, Montenegro-Burke JR, Domingo-Almenara X, et al. 2018 METLIN: a technology platform for identifying knowns and unknowns. Analytical Chemistry 90, 3156–3164. 29381867 10.1021/acs.analchem.7b04424 PMC5933435

[CIT0042] Guo Gao T, Yuan Xu Y, Jiang F, Zhen Li B, Shui Yang J, Tao Wang E, Li Yuan H 2015 Nodulation characterization and proteomic profiling of *Bradyrhizobium liaoningense CCBAU05525* in response to water-soluble humic materials. Scientific Reports 5, 10836. 26054030 10.1038/srep10836 PMC4650689

[CIT0043] Harris D, Breese WA, Rao JVDKK 2005 The improvement of crop yield in marginal environments using on-farm seed priming: nodulation, nitrogen fixation, and disease resistance. Australian Journal of Agricultural Research 56, 1211–1218.

[CIT0044] Hayashi S, Reid DE, Lorenc MT, Stiller J, Edwards D, Gresshoff PM, Ferguson BJ 2012 Transient *Nod* factor-dependent gene expression in the nodulation-competent zone of soybean (*Glycine max* [L.] Merr.) roots. Plant Biotechnology Journal 10, 995–1010. 22863334 10.1111/j.1467-7652.2012.00729.x

[CIT0045] Iannetta PP, Young M, Bachinger J, et al. 2016 A comparative nitrogen balance and productivity analysis of legume and non-legume supported cropping systems: the potential role of biological nitrogen fixation. Frontiers in Plant Science 7, 1700. 27917178 10.3389/fpls.2016.01700 PMC5116563

[CIT0046] Indrasumunar A, Searle I, Lin MH, Kereszt A, Men A, Carroll BJ, Gresshoff PM 2011 Nodulation factor receptor kinase 1α controls nodule organ number in soybean (*Glycine max* L. Merr). The Plant Journal 65, 39–50. 21175888 10.1111/j.1365-313X.2010.04398.x

[CIT0047] Kant C, Pradhan S, Bhatia S 2016 Dissecting the root nodule transcriptome of chickpea (*Cicer arietinum* L.). PLoS One 11, e0157908. 27348121 10.1371/journal.pone.0157908 PMC4922567

[CIT0048] Kaschani F, Nickel S, Pandey B, Cravatt BF, Kaiser M, van der Hoorn RA 2012 Selective inhibition of plant serine hydrolases by agrochemicals revealed by competitive ABPP. Bioorganic & Medicinal Chemistry 20, 597–600. 21764588 10.1016/j.bmc.2011.06.040 PMC3634566

[CIT0049] Kawaharada Y, Kelly S, Nielsen MW, et al. 2015 Receptor-mediated exopolysaccharide perception controls bacterial infection. Nature 523, 308–312. 26153863 10.1038/nature14611

[CIT0050] Knogge W, Scheel D 2006 LysM receptors recognize friend and foe. Proceedings of the National Academy of Sciences, USA 103, 10829–10830. 10.1073/pnas.0604601103 PMC1544133 16832046

[CIT0051] Kouchi H, Hata S 1993 Isolation and characterization of novel *nodulin* cDNAs representing genes expressed at early stages of soybean nodule development. Molecular & General Genetics 238, 106–119. 7683079 10.1007/BF00279537

[CIT0052] Kouchi H, Shimomura K, Hata S, et al. 2004 Large-scale analysis of gene expression profiles during early stages of root nodule formation in a model legume, *Lotus japonicus*. DNA Research 11, 263–274. 15500251 10.1093/dnares/11.4.263

[CIT0053] Kriventseva EV, Zdobnov EM, Simão FA, Ioannidis P, Waterhouse RM 2015 BUSCO: assessing genome assembly and annotation completeness with single-copy orthologs. Bioinformatics 31, 3210–3212. 26059717 10.1093/bioinformatics/btv351

[CIT0054] Krusell L, Madsen LH, Sato S, et al. 2002 Shoot control of root development and nodulation is mediated by a receptor-like kinase. Nature 420, 422–426. 12442170 10.1038/nature01207

[CIT0055] Lafuente A, Pajuelo E, Caviedes MA, Rodríguez-Llorente ID 2010 Reduced nodulation in alfalfa induced by arsenic correlates with altered expression of early nodulins. Journal of Plant Physiology 167, 286–291. 19879664 10.1016/j.jplph.2009.09.014

[CIT0056] Lamar R, Olk DC, Mayhew L, Bloom PR 2013 Evaluation of a proposed standardized analytical method for the determination of humic and fulvic acids in commercial products. In: Xu J, Wu J, He Y, eds. Functions of natural organic matter in changing environment. Dordrecht: Springer, 1071–1073.

[CIT0057] Larrainzar E, Riely BK, Kim SC, et al. 2015 Deep sequencing of the *Medicago truncatula* root transcriptome reveals a massive and early interaction between nodulation factor and ethylene signals. Plant Physiology 169, 233–265. 26175514 10.1104/pp.15.00350 PMC4577383

[CIT0058] Law CW, Chen Y, Shi W, Smyth GK 2014 voom: precision weights unlock linear model analysis tools for RNA-seq read counts. Genome Biology 15, R29. 24485249 10.1186/gb-2014-15-2-r29 PMC4053721

[CIT0059] Ledgard SF, Steele KW 1992 Biological nitrogen fixation in mixed legume/grass pastures. Plant and Soil 141, 137–153.

[CIT0060] Legocki RP, Verma DP 1980 Identification of ‘nodule-specific’ host proteins (nodulins) involved in the development of *Rhizobium*–legume symbiosis. Cell 20, 153–163. 7388942 10.1016/0092-8674(80)90243-3

[CIT0061] Liang Y, Tóth K, Cao Y, Tanaka K, Espinoza C, Stacey G 2014 Lipochitooligosaccharide recognition: an ancient story. New Phytologist 204, 289–296. 25453133 10.1111/nph.12898

[CIT0062] Libault M 2014 The carbon–nitrogen balance of the nodule and its regulation under elevated carbon dioxide concentration. BioMed Research International 2014, 507946. 24987690 10.1155/2014/507946 PMC4058508

[CIT0063] Libault M, Farmer A, Joshi T, Takahashi K, Langley RJ, Franklin LD, He J, Xu D, May G, Stacey G 2010 An integrated transcriptome atlas of the crop model *Glycine max*, and its use in comparative analyses in plants. The Plant Journal 63, 86–99. 20408999 10.1111/j.1365-313X.2010.04222.x

[CIT0064] Lim CW, Lee YW, Hwang CH 2011 Soybean nodule-enhanced CLE peptides in roots act as signals in *GmNARK*-mediated nodulation suppression. Plant & Cell Physiology 52, 1613–1627. 21757457 10.1093/pcp/pcr091

[CIT0065] Limpens E, Franken C, Smit P, Willemse J, Bisseling T, Geurts R 2003 LysM domain receptor kinases regulating rhizobial Nod factor-induced infection. Science 302, 630–633. 12947035 10.1126/science.1090074

[CIT0066] Linstrom PJ, Mallard WG, **eds.** 2018 NIST Chemistry WebBook, NIST Standard Reference Database Number 69. Gaithersburg, MD: National Institute of Standards and Technology.

[CIT0067] Little K, Rose M, Patti A, Cavagnaro T, Jackson R 2013 Effect of application rate of commercial lignite coal-derived amendments on early-stage growth of *Medicago sativa* and soil health, in acidic soil conditions. In: Xu J, Wu J, He Y, eds. Functions of natural organic matter in changing environment. Dordrecht: Springer, 1085–1088.

[CIT0068] Little KR, Rose MT, Jackson WR, Cavagnaro TR, Patti AF 2014 Do lignite-derived organic amendments improve early-stage pasture growth and key soil biological and physicochemical properties? Crop and Pasture Science 65, 899–910.

[CIT0069] Liu A, Contador CA, Fan K, Lam HM 2018 Interaction and regulation of carbon, nitrogen, and phosphorus metabolisms in root nodules of legumes. Frontiers in Plant Science 9, 1860. 30619423 10.3389/fpls.2018.01860 PMC6305480

[CIT0070] Liu Y-C, Lei Y-W, Liu W, Weng L, Lei M-J, Hu X-H, Dong Z, Luo D, Yang J 2018 LjCOCH interplays with LjAPP1 to maintain the nodule development in *Lotus japonicus*. Plant Growth Regulation 85, 267–279.

[CIT0071] Livak KJ, Schmittgen TD 2001 Analysis of relative gene expression data using real-time quantitative PCR and the 2^–ΔΔCT^ method. Methods 25, 402–408. 11846609 10.1006/meth.2001.1262

[CIT0072] Lohar DP, Sharopova N, Endre G, Peñuela S, Samac D, Town C, Silverstein KA, VandenBosch KA 2006 Transcript analysis of early nodulation events in *Medicago truncatula*. Plant Physiology 140, 221–234. 16377745 10.1104/pp.105.070326 PMC1326046

[CIT0073] Lüscher A, Mueller-Harvey I, Soussana JF, Rees RM, Peyraud JL 2014 Potential of legume-based grassland–livestock systems in Europe: a review. Grass and Forage Science 69, 206–228. 26300574 10.1111/gfs.12124 PMC4540161

[CIT0074] Madsen EB, Madsen LH, Radutoiu S, et al. 2003 A receptor kinase gene of the *LysM* type is involved in legume perception of rhizobial signals. Nature 425, 637–640. 14534591 10.1038/nature02045

[CIT0075] Maji D, Misra P, Singh S, Kalra A 2017 Humic acid rich vermicompost promotes plant growth by improving microbial community structure of soil as well as root nodulation and mycorrhizal colonization in the roots of *Pisum sativum*. Applied Soil Ecology 110, 97–108.

[CIT0076] Marguerat S, Bähler J 2010 RNA-seq: from technology to biology. Cellular and Molecular Life Sciences 67, 569–579. 19859660 10.1007/s00018-009-0180-6 PMC2809939

[CIT0077] Marsh JF, Rakocevic A, Mitra RM, Brocard L, Sun J, Eschstruth A, Long SR, Schultze M, Ratet P, Oldroyd GE 2007 *Medicago truncatula NIN* is essential for rhizobial-independent nodule organogenesis induced by autoactive calcium/calmodulin-dependent protein kinase. Plant Physiology 144, 324–335. 17369436 10.1104/pp.106.093021 PMC1913781

[CIT0078] Martin LB, Fei Z, Giovannoni JJ, Rose JK 2013 Catalyzing plant science research with RNA-seq. Frontiers in Plant Science 4, 66. 23554602 10.3389/fpls.2013.00066 PMC3612697

[CIT0079] Mathers NJ, Jalota RK, Dalal RC, Boyd SE 2007 ^13^C-NMR analysis of decomposing litter and fine roots in the semi-arid Mulga Lands of southern Queensland. Soil Biology and Biochemistry 39, 993–1006.

[CIT0080] Mathers NJ, Xu Z 2003 Solid-state ^13^C NMR spectroscopy: characterization of soil organic matter under two contrasting residue management regimes in a 2-year-old pine plantation of subtropical Australia. Geoderma 114, 19–31.

[CIT0081] Mathis R, Grosjean C, de Billy F, Huguet T, Gamas P 1999 The early nodulin gene *MtN6* is a novel marker for events preceding infection of *Medicago truncatula* roots by *Sinorhizobium meliloti*. Molecular Plant-Microbe Interactions 12, 544–555. 10356802 10.1094/MPMI.1999.12.6.544

[CIT0082] McLoughlin AJ, Küster E 1972 The effect of humic substances on the respiration and growth of micro-organisms. Plant and Soil 37, 17–25.

[CIT0083] Mi H, Dong Q, Muruganujan A, Gaudet P, Lewis S, Thomas PD 2010 PANTHER version 7: improved phylogenetic trees, orthologs and collaboration with the Gene Ontology Consortium. Nucleic Acids Research 38, D204–D210. 20015972 10.1093/nar/gkp1019 PMC2808919

[CIT0084] Middleton PH, Jakab J, Penmetsa RV, et al. 2007 An ERF transcription factor in *Medicago truncatula* that is essential for Nod factor signal transduction. The Plant Cell 19, 1221–1234. 17449807 10.1105/tpc.106.048264 PMC1913751

[CIT0085] Mindrebo JT, Nartey CM, Seto Y, Burkart MD, Noel JP 2016 Unveiling the functional diversity of the alpha/beta hydrolase superfamily in the plant kingdom. Current Opinion in Structural Biology 41, 233–246. 27662376 10.1016/j.sbi.2016.08.005 PMC5687975

[CIT0086] Mitchell AL, Attwood TK, Babbitt PC, et al. 2019 InterPro in 2019: improving coverage, classification and access to protein sequence annotations. Nucleic Acids Research 47, D351–D360. 30398656 10.1093/nar/gky1100 PMC6323941

[CIT0087] Moreau S, Verdenaud M, Ott T, Letort S, de Billy F, Niebel A, Gouzy J, de Carvalho-Niebel F, Gamas P 2011 Transcription reprogramming during root nodule development in *Medicago truncatula*. PLoS One 6, e16463. 21304580 10.1371/journal.pone.0016463 PMC3029352

[CIT0088] Mortazavi A, Williams BA, McCue K, Schaeffer L, Wold B 2008 Mapping and quantifying mammalian transcriptomes by RNA-Seq. Nature Methods 5, 621–628. 18516045 10.1038/nmeth.1226 PMC13303166

[CIT0089] Mortier V, Den Herder G, Whitford R, Van de Velde W, Rombauts S, D’Haeseleer K, Holsters M, Goormachtig S 2010 CLE peptides control *Medicago truncatula* nodulation locally and systemically. Plant Physiology 153, 222–237. 20348212 10.1104/pp.110.153718 PMC2862434

[CIT0090] Muñoz N, Soria-Díaz ME, Manyani H, Sánchez-Matamoros RC, Serrano AG, Megías M, Lascano R 2014 Structure and biological activities of lipochitooligosaccharide nodulation signals produced by *Bradyrhizobium japonicum* USDA 138 under saline and osmotic stress. Biology and Fertility of Soils 50, 207–215.

[CIT0091] Nakagawa T, Kaku H, Shimoda Y, Sugiyama A, Shimamura M, Takanashi K, Yazaki K, Aoki T, Shibuya N, Kouchi H 2011 From defense to symbiosis: limited alterations in the kinase domain of LysM receptor-like kinases are crucial for evolution of legume–*Rhizobium* symbiosis. The Plant Journal 65, 169–180. 21223383 10.1111/j.1365-313X.2010.04411.x

[CIT0092] Nardi S, Panuccio MR, Abenavoli MR, Muscolo A 1994 Auxin-like effect of humic substances extracted from faeces of *Allolobophora caliginosa* and *A. rosea*. Soil Biology and Biochemistry 26, 1341–1346.

[CIT0093] Nardi S, Pizzeghello D, Muscolo A, Vianello A 2002 Physiological effects of humic substances on higher plants. Soil Biology and Biochemistry 34, 1527–1536.

[CIT0094] NCBI Resource Coordinators 2016 Database resources of the National Center for Biotechnology Information. Nucleic Acids Research 44, D7–D19. 26615191 10.1093/nar/gkv1290 PMC4702911

[CIT0095] O’Rourke JA, Yang SA, Miller SS, et al 2013 An RNA-Seq transcriptome analysis of orthophosphate-deficient white lupin reveals novel insights into phosphorus acclimation in plants. Plant Physiology 161, 705–724. 23197803 10.1104/pp.112.209254 PMC3561014

[CIT0096] Oka-Kira E, Kawaguchi M 2006 Long-distance signaling to control root nodule number. Current Opinion in Plant Biology 9, 496–502. 16877028 10.1016/j.pbi.2006.07.012

[CIT0097] Oldroyd GE 2013 Speak, friend, and enter: signalling systems that promote beneficial symbiotic associations in plants. Nature Reviews. Microbiology 11, 252–263. 23493145 10.1038/nrmicro2990

[CIT0098] Pandeya SB, Singh AK, Dhar P 1998 Influence of fulvic acid on transport of iron in soils and uptake by paddy seedlings. Plant and Soil 198, 117–125.

[CIT0099] Peng Z, Liu F, Wang L, Zhou H, Paudel D, Tan L, Maku J, Gallo M, Wang J 2017 Transcriptome profiles reveal gene regulation of peanut (*Arachis hypogaea* L.) nodulation. Scientific Reports 7, 40066. 28059169 10.1038/srep40066 PMC5216375

[CIT0100] Pinto AP, Mota AM, de Varennes A, Pinto FC 2004 Influence of organic matter on the uptake of cadmium, zinc, copper and iron by sorghum plants. The Science of the Total Environment 326, 239–247. 15142779 10.1016/j.scitotenv.2004.01.004

[CIT0101] Popp C, Ott T 2011 Regulation of signal transduction and bacterial infection during root nodule symbiosis. Current Opinion in Plant Biology 14, 458–467. 21489860 10.1016/j.pbi.2011.03.016

[CIT0102] Powell DR 2015 Degust: visualize, explore and appreciate RNA-seq differential gene-expression data. http://victorian-bioinformatics-consortium.github.io/degust/

[CIT0103] Preissel S, Reckling M, Schläfke N, Zander P 2015 Magnitude and farm-economic value of grain legume pre-crop benefits in Europe: a review. Field Crops Research 175, 64–79.

[CIT0104] Pringle D, Dickstein R 2004 Purification of ENOD8 proteins from *Medicago sativa* root nodules and their characterization as esterases. Plant Physiology and Biochemistry 42, 73–79. 15061087 10.1016/j.plaphy.2003.10.004

[CIT0105] Pruitt KD, Tatusova T, Maglott DR 2005 NCBI Reference Sequence (RefSeq): a curated non-redundant sequence database of genomes, transcripts and proteins. Nucleic Acids Research 33, D501–D504. 15608248 10.1093/nar/gki025 PMC539979

[CIT0106] Radutoiu S, Madsen LH, Madsen EB, et al. 2003 Plant recognition of symbiotic bacteria requires two LysM receptor-like kinases. Nature 425, 585–592. 14534578 10.1038/nature02039

[CIT0107] Reckling M, Hecker J-M, Bergkvist G, et al 2016 A cropping system assessment framework—evaluating effects of introducing legumes into crop rotations. European Journal of Agronomy 76, 186–197.

[CIT0108] Reddy PM, Ladha JK, Ramos MC, Maillet F, Hernandez RJ, Torrizo LB, Oliva NP, Datta SK, Datta K 1998 Rhizobial lipochitooligosaccharide nodulation factors activate expression of the legume early nodulin gene *ENOD12* in rice. The Plant Journal 14, 693–702.

[CIT0109] Reid DE, Ferguson BJ, Gresshoff PM 2011 Inoculation- and nitrate-induced CLE peptides of soybean control *NARK*-dependent nodule formation. Molecular Plant-Microbe Interactions 24, 606–618. 21198362 10.1094/MPMI-09-10-0207

[CIT0110] Rivers RL, Dean RM, Chandy G, Hall JE, Roberts DM, Zeidel ML 1997 Functional analysis of nodulin 26, an aquaporin in soybean root nodule symbiosomes. Journal of Biological Chemistry 272, 16256–16261. 9195927 10.1074/jbc.272.26.16256

[CIT0111] Roberts DM, Routray P 2017 The nodulin 26 intrinsic protein subfamily. In: Chaumont F, Tyerman SD, eds. Plant aquaporins: from transport to signaling. Cham: Springer International Publishing, 267–296.

[CIT0112] Roux B, Rodde N, Jardinaud MF, et al. 2014 An integrated analysis of plant and bacterial gene expression in symbiotic root nodules using laser-capture microdissection coupled to RNA sequencing. The Plant Journal 77, 817–837. 24483147 10.1111/tpj.12442

[CIT0113] Russell L, Stokes AR, Macdonald H, Muscolo A, Nardi S 2006 Stomatal responses to humic substances and auxin are sensitive to inhibitors of phospholipase A2. Plant and Soil 283, 175–185.

[CIT0114] Scheres B, van Engelen F, van der Knaap E, van de Wiel C, van Kammen A, Bisseling T 1990 Sequential induction of *nodulin* gene expression in the developing pea nodule. The Plant Cell 2, 687–700. 2152123 10.1105/tpc.2.8.687 PMC159922

[CIT0115] Schirawski J, Perlin MH 2018 Plant–microbe interaction 2017—the good, the bad and the diverse. International Journal of Molecular Sciences 19, 1374. 10.3390/ijms19051374 PMC5983726 29734724

[CIT0116] Schnabel E, Journet EP, de Carvalho-Niebel F, Duc G, Frugoli J 2005 The *Medicago truncatula SUNN* gene encodes a CLV1-like leucine-rich repeat receptor kinase that regulates nodule number and root length. Plant Molecular Biology 58, 809–822. 16240175 10.1007/s11103-005-8102-y

[CIT0117] Singleton PW, Tavares JW 1986 Inoculation response of legumes in relation to the number and effectiveness of indigenous *Rhizobium* populations. Applied and Environmental Microbiology 51, 1013–1018. 16347046 10.1128/aem.51.5.1013-1018.1986 PMC239003

[CIT0118] Smith CA, O’Maille G, Want EJ, Qin C, Trauger SA, Brandon TR, Custodio DE, Abagyan R, Siuzdak G 2005 METLIN: a metabolite mass spectral database. Therapeutic Drug Monitoring 27, 747–751. 16404815 10.1097/01.ftd.0000179845.53213.39

[CIT0119] Sutton R, Sposito G 2005 Molecular structure in soil humic substances: the new view. Environmental Science & Technology 39, 9009–9015. 16382919 10.1021/es050778q

[CIT0120] Tan KH, Tantiwiramanond D 1983 Effect of humic acids on nodulation and dry matter production of soybean, peanut, and clover. Soil Science Society of America 47, 1121–1124.

[CIT0121] The Gene Ontology Consortium 2019 The Gene Ontology Resource: 20 years and still GOing strong. Nucleic Acids Research 47, D330–D338. 30395331 10.1093/nar/gky1055 PMC6323945

[CIT0122] Thies JE, Singleton PW, Bohlool BB 1991 Influence of the size of indigenous rhizobial populations on establishment and symbiotic performance of introduced rhizobia on field-grown legumes. Applied and Environmental Microbiology 57, 19–28. 16348393 10.1128/aem.57.1.19-28.1991 PMC182659

[CIT0123] ‘t Hoen PA, Ariyurek Y, Thygesen HH, Vreugdenhil E, Vossen RH, de Menezes RX, Boer JM, van Ommen GJ, den Dunnen JT 2008 Deep sequencing-based expression analysis shows major advances in robustness, resolution and inter-lab portability over five microarray platforms. Nucleic Acids Research 36, e141. 18927111 10.1093/nar/gkn705 PMC2588528

[CIT0124] Thomas PD, Campbell MJ, Kejariwal A, Mi H, Karlak B, Daverman R, Diemer K, Muruganujan A, Narechania A 2003 PANTHER: a library of protein families and subfamilies indexed by function. Genome Research 13, 2129–2141. 12952881 10.1101/gr.772403 PMC403709

[CIT0125] Thomas PD, Kejariwal A, Guo N, Mi H, Campbell MJ, Muruganujan A, Lazareva-Ulitsky B 2006 Applications for protein sequence–function evolution data: mRNA/protein expression analysis and coding SNP scoring tools. Nucleic Acids Research 34, W645–W650. 16912992 10.1093/nar/gkl229 PMC1538848

[CIT0126] Traversa A, Loffredo E, Gattullo CE, Palazzo AJ, Bashore TL, Senesi N 2014 Comparative evaluation of compost humic acids and their effects on the germination of switchgrass (*Panicum vigatum* L.). Journal of Soils and Sediments 14, 432–440.

[CIT0127] Trevisan S, Francioso O, Quaggiotti S, Nardi S 2010 *a* Humic substances biological activity at the plant–soil interface: from environmental aspects to molecular factors. Plant Signaling & Behavior 5, 635–643. 20495384 10.4161/psb.5.6.11211 PMC3001551

[CIT0128] Trevisan S, Pizzeghello D, Ruperti B, Francioso O, Sassi A, Palme K, Quaggiotti S, Nardi S 2010 *b* Humic substances induce lateral root formation and expression of the early auxin-responsive *IAA19* gene and *DR5* synthetic element in *Arabidopsis*. Plant Biology 12, 604–614. 20636903 10.1111/j.1438-8677.2009.00248.x

[CIT0129] UniProt Consortium 2018 UniProt: a worldwide hub of protein knowledge. Nucleic Acids Research 47, D506–D515. 10.1093/nar/gky1049 PMC6323992 30395287

[CIT0130] Vaccaro S, Ertani A, Nebbioso A, Muscolo A, Quaggiotti S, Piccolo A, Nardi S 2015 Humic substances stimulate maize nitrogen assimilation and amino acid metabolism at physiological and molecular level. Chemical and Biological Technologies in Agriculture 2, 5.

[CIT0131] van de Wiel C, Scheres B, Franssen H, van Lierop MJ, van Lammeren A, van Kammen A, Bisseling T 1990 The early nodulin transcript *ENOD2* is located in the nodule parenchyma (inner cortex) of pea and soybean root nodules. EMBO Journal 9, 1–7. 1688528 10.1002/j.1460-2075.1990.tb08073.x PMC551621

[CIT0132] Verlinden G, Coussens T, De Vliegher A, Baert G, Haesaert G 2010 Effect of humic substances on nutrient uptake by herbage and on production and nutritive value of herbage from sown grass pastures. Grass and Forage Science 65, 133–144.

[CIT0133] Vernié T, Moreau S, de Billy F, Plet J, Combier JP, Rogers C, Oldroyd G, Frugier F, Niebel A, Gamas P 2008 *EFD* is an *ERF* transcription factor involved in the control of nodule number and differentiation in *Medicago truncatula*. The Plant Cell 20, 2696–2713. 18978033 10.1105/tpc.108.059857 PMC2590733

[CIT0134] Visser SA 1985 Physiological action of humic substances on microbial cells. Soil Biology and Biochemistry 17, 457–462.

[CIT0135] Wang X, Chen X, Wang Z, Nikolay D, Vladimir C, Gao H 2010 Isolation and characterization of *GoDREB* encoding an ERF-type protein in forage legume *Galegae orientalis*. Genes & Genetic Systems 85, 157–166. 21041975 10.1266/ggs.85.157

[CIT0136] Wang Z, Gerstein M, Snyder M 2009 RNA-Seq: a revolutionary tool for transcriptomics. Nature Reviews. Genetics 10, 57–63. 10.1038/nrg2484 PMC2949280 19015660

[CIT0137] Xu YY, Yang JS, Liu C, Wang ET, Wang RN, Qiu XQ, Li BZ, Chen WF, Yuan HL 2018 Water-soluble humic materials regulate quorum sensing in *Sinorhizobium meliloti* through a novel repressor of expR. Frontiers in Microbiology 9, 3194. 30627123 10.3389/fmicb.2018.03194 PMC6309736

[CIT0138] Zaretskaya I, Johnson M, McGinnis S, Raytselis Y, Merezhuk Y, Madden TL 2008 NCBI BLAST: a better web interface. Nucleic Acids Research 36, W5–W9. 18440982 10.1093/nar/gkn201 PMC2447716

[CIT0139] Zeijl Av, Guhl K, Xiao TT, Shen D, Geurts R, Kohlen W 2018 Nitrate inhibition of nodule formation in *Medicago truncatula* is mediated by *ACC SYNTHASE 10*. bioRxiv 434829. [Preprint].

[CIT0140] Zhang X, Zhang X, Liu X, Shao L, Sun H, Chen S 2015 Improving winter wheat performance by foliar spray of ABA and FA under water deficit conditions. Journal of Plant Growth Regulation 35, 83–96.

[CIT0141] Zherebker A, Shirshin E, Kharybin O, et al. 2018 Separation of benzoic and unconjugated acidic components of leonardite humic material using sequential solid-phase extraction at different pH values as revealed by Fourier transform ion cyclotron resonance mass spectrometry and correlation nuclear magnetic resonance spectroscopy. Journal of Agricultural and Food Chemistry 66, 12179–12187. 30335379 10.1021/acs.jafc.8b04079

[CIT0142] Zhimang G, Xiaorong W, Xueyuan G, Jing C, Liansheng W, Lemei D, Yijun C 2001 Effects of fulvic acid on the bioavailability of rare earth elements and GOT enzyme activity in wheat (*Triticum aestivum*). Chemosphere 44, 545–551. 11482641 10.1016/s0045-6535(00)00484-7

[CIT0143] Zipfel C, Oldroyd GE 2017 Plant signalling in symbiosis and immunity. Nature 543, 328–336. 28300100 10.1038/nature22009

