# Supplementary material for: Fulvic acid increases forage legume growth inducing preferential up-regulation of nodulation and signalling-related genes
Source: J Exp Bot. 2020 Jun 30;71(18):5689–704. doi: 10.1093/jxb/eraa283 (PMC7501823; doi:10.1093/jxb/eraa283)
Supplement: eraa283_suppl_Supplementary_Figures_and_Tables [file eraa283_suppl_supplementary_figures_and_tables.pdf]

Supplementary tables and figures.

Fulvic acid increases forage legume growth inducing preferential upregulation of nodulation and signalling-related genes. Capstaff et al. 2020.

Supplementary Table S1. MFA detectable content in mg/L for individual elements by ICP-OES and ICP-MS for 0.5 % solution.

Ti	Mo	Cr	Cd	P	Zn
337.280	202.032	267.716	226.502	213.618	213.857
$6.18e^{-2}$	$3.57e^{-4}$	$4.86e^{-4}$	$1.25e^{-4}$	$4.24e^{-2}$	$4.43e^{-3}$
Co	Ni	Cu	Fe	Mn	Total N
228.615	231.604	324.754	259.940	259.372	
$1.06e^{-4}$	$2.54e^{-4}$	$1.13e^{-3}$	2.53	$1.13e^{-3}$	8.59

Supplementary Table S2. VFA detectable content in mg/L for individual elements by ICP-OES and ICP-MS for 0.5 % solution.

Al	Ca	Co	Cu	Fe	K	Mg
396.153	315.887	59	63	238.204	766.490	279.077
$4.33e^{-1}$	19.06	$2.55e^{-3}$	$7.49e^{-3}$	$4.20e^{-1}$	1.72	8.90
Mn	Ni	S	Zn	Na	Total N	Total C
55	60	181.975	66	589.592		
$1.07e^{-1}$	$5.95e^{-3}$	37.11	$3.44e^{-2}$	15.90	1.51	0.03

Supplementary Table S3. MFA control solution (MC) components for 0.5 % application; compound is given, with Sigma-Aldrich catalogue number, and mg/L required.

NH_4NO_3	$CuSO_4{\cdot}5H_2O$	$FeSO_4.7H_2O$	Monosodium citrate
A7455	C8027	F8263	71498
24.5	8	12.45	45.6

pH to 4.8

Supplementary Table S4. VFA control solution (VC) components for 1.0 % application; compound is given, with Sigma-Aldrich catalogue number, and mg/L required.

KNO_3	Sucrose	$AlCl_3$	$CaSO_4.2H_2O$	$FeSO_4.7H_2O$	K_2SO_4
P8291	84097	563919	C3771	F8263	P0772
1.95	125.5	7.75	163.7	4.2	13.2
$MgSO_4$	$MnSO_4.xH_2O$	$Zn(NO_3)_2$.H2O	NaCl	Na_2SO_4	PEG-400
M7506	229784	230006	S7653	S6547	202938
88.15	0.65	0.15	6.4	74.4	62.75

pH to 6.0

Supplementary Tables S5 to S11 in Excel file.

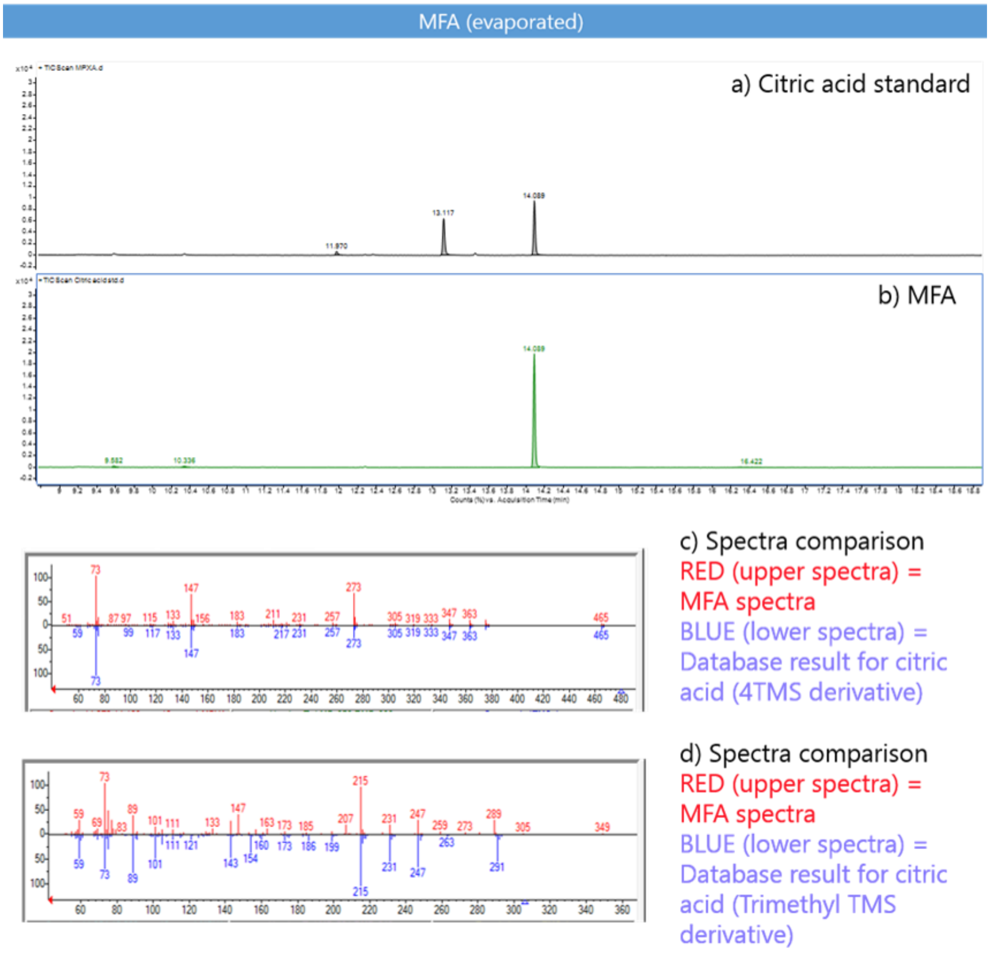

Supplementary Fig. S1. Gas chromatogram spectra of (a) citric acid standard and b) MFA, with c)-d) NIST Atomic Spectra database 1A v14 matches for citric acid, 4TMS derivative and Trimethyl TMS derivative; both citric acid monohydrate standard and MFA at a concentration of 0.01 g/mL.

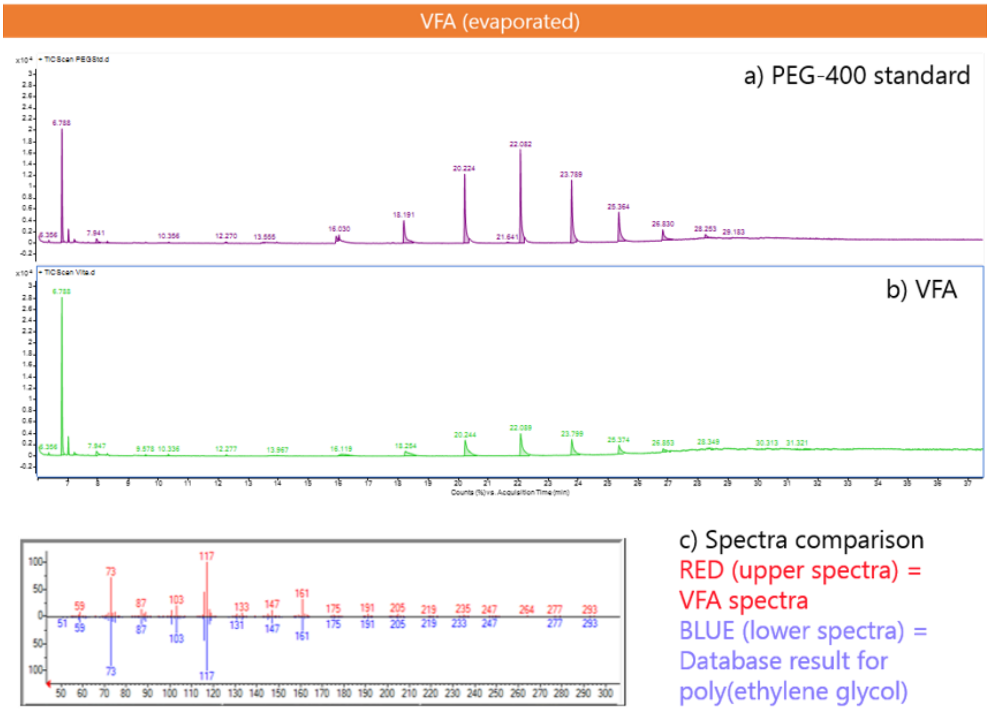

Supplementary Fig. S2. Gas chromatogram spectra of (a) PEG-400 standard and b) VFA, with c) NIST Atomic Spectra database 1A v14 matches for poly(ethylene glycol) (heptaethylene glycol); both poly(ethylene glycol)-400 standard and VFA at a concentration of 0.01 g/mL.

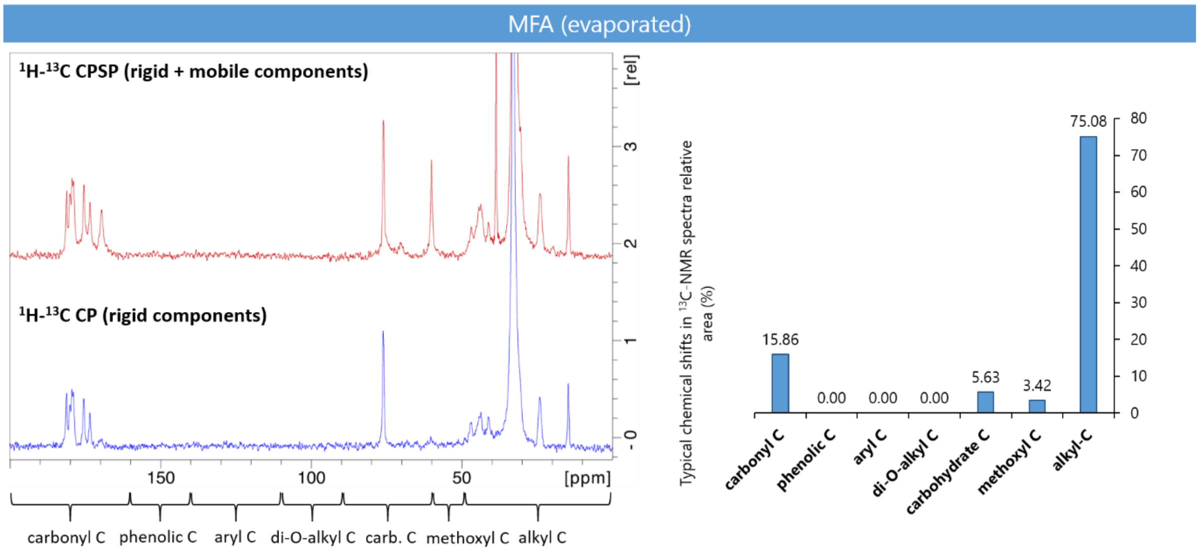

Supplementary Fig. S3. MFA NMR analysis. (Left) ^{1}H-^{13}C CP (blue spectrum, rigid components) and CPSP (red spectrum, rigid + mobile components) NMR spectra of evaporated MFA. (Right) Bar graph showing the composition of functional groups, in percentage, obtained from spectral analysis of the ^{1}H-^{13}C CPSP spectrum of evaporated MFA (left, blue). The values are specified on top of each bar.

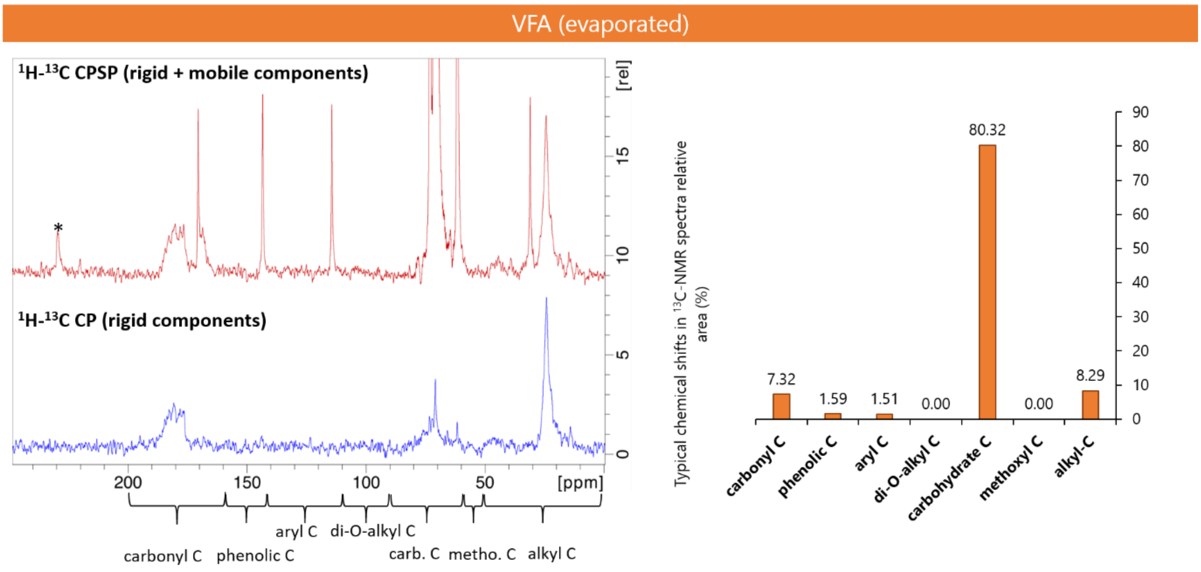

Supplementary Fig. S4: VFA NMR analysis. (Left) ^{1}H-^{13}C CP (blue spectrum, rigid components) and CPSP (red spectrum, rigid + mobile components) NMR spectra of evaporated VFA. (Right) Bar graph showing the composition of functional groups, in percentage, obtained from spectral analysis of the ^{1}H-^{13}C CPSP spectrum of evaporated VFA (left, blue). The values are specified on top of each bar. ('*' is an unknown peak in VFA CPSP spectrum and is not included in the composition analysis).

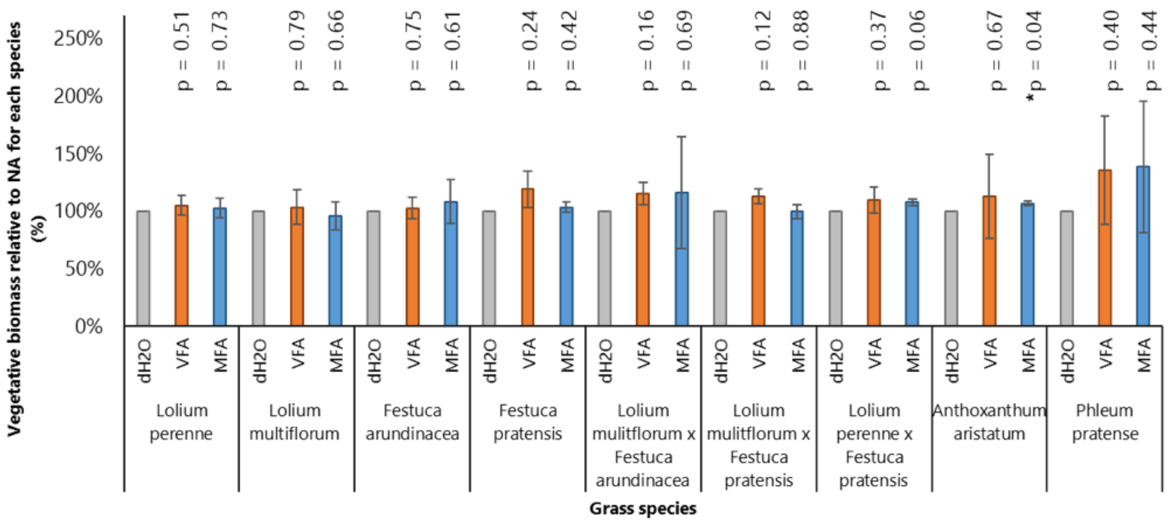

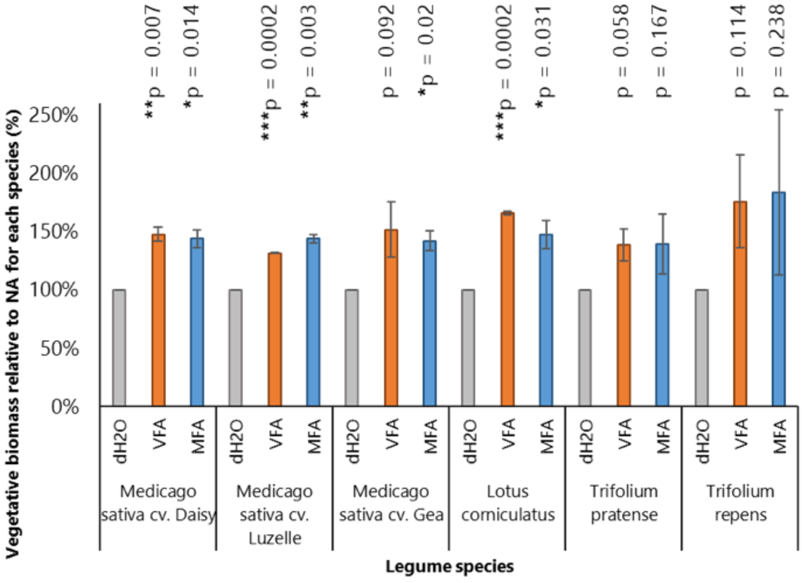

Supplementary Fig. S5. Vegetative biomass of forage crops following one of two fulvic acid treatments relative to a dH2O. Treatments were applied to seedlings at 7 days post germination and vegetative yields were assessed at 21 days post treatment. Treatments were deionised water (dH_2O in grey), 0.5 % MFA (blue), or 1 % VFA (orange). Fifteen forage crop species/cultivars were tested in total, with the top bar chart showing grass species, and the below bar chart showing legumes. Biomass was measured for two independent experiments with biomass for both FAs calculated relative to dH_2O (shown as 100%). Error bars show standard deviation between experiments. Two-tailed student t-tests were calculated between dH_2O and each FA and are indicated in graphs; those with p-value < 0.05 are indicated with '*', with p-value < 0.01 with '**', and with p-value < 0.001 with '***'.

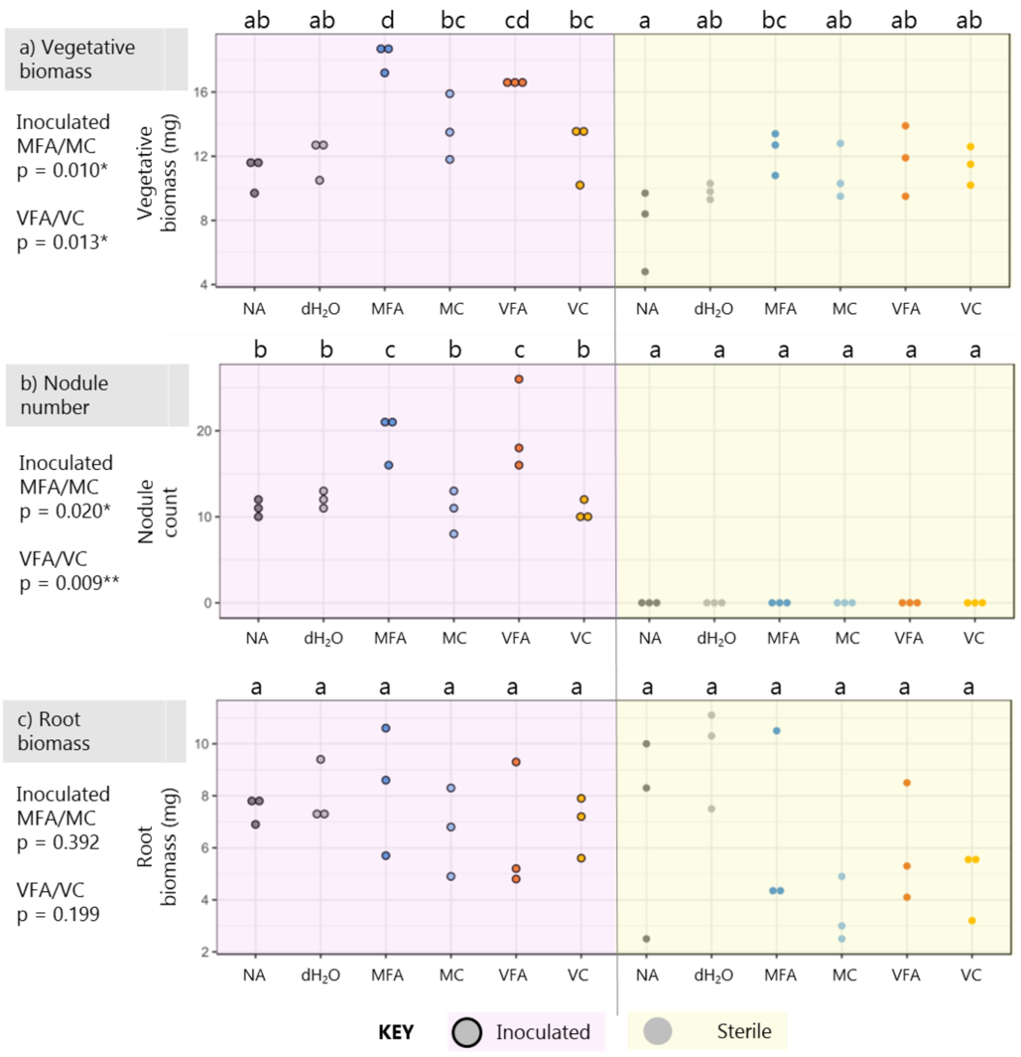

Supplementary Fig. S6. Vegetative and nodule phenotypes of plate grown *Medicago sativa* cv. Daisy following treatment with fulvic acids or controls, with or without inoculation of *Sinorhizobium meliloti*. Two day old seedlings were transferred to media plates containing treatments as follows; no addition (NA in dark grey); deionised water (dH_2O in grey); 0.5 % MFA (MFA in blue); 0.5 % MC (MC in light blue); 1 % VFA (VFA in orange); 1 % VC (VC in yellow). Plates either remained sterile (open dots on yellow background) or inoculated with *Sinorhizobium meliloti* (closed dots on purple). At 21 days the nodule numbers were counted, and biomass determined for both vegetative tissue and full root tissue. Five seedlings were measured for each treatment condition, and total measurements for three independent experiments are shown in charts. Multiple comparisons were conducted using a one-way ANOVA Tukey test shown with letters, and one-tailed student t-tests were calculated for FAs and elemental controls with p-value significance indicated for inoculated plates only.

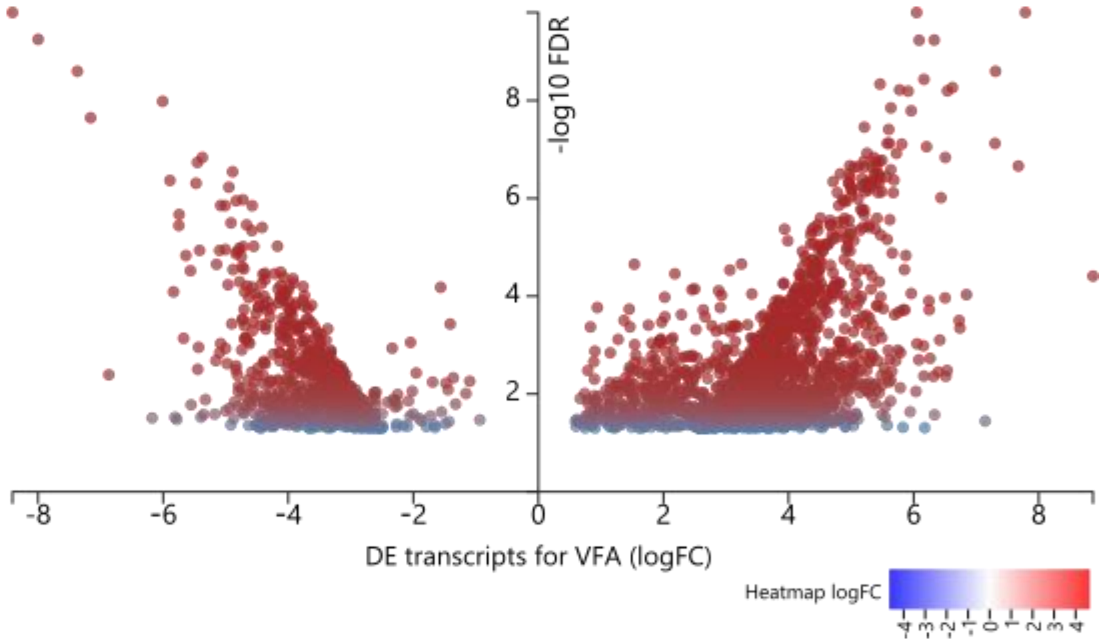

Supplementary Fig. S7. Volcano plot of DE transcripts as log Fold Change (logFC) between 0 day and 3 day for VFA treatment RNA samples; Root tissue data is shown for *de novo* RPKM sample comparisons and transcripts are deemed DE if all experimental replicates have an absolute log fold change of 0.585 and false-discovery rate adjusted q-value < 0.05; graph modified from Degust (Powell, 2014) and made using voom/Limma method (Law *et al.*, 2014).

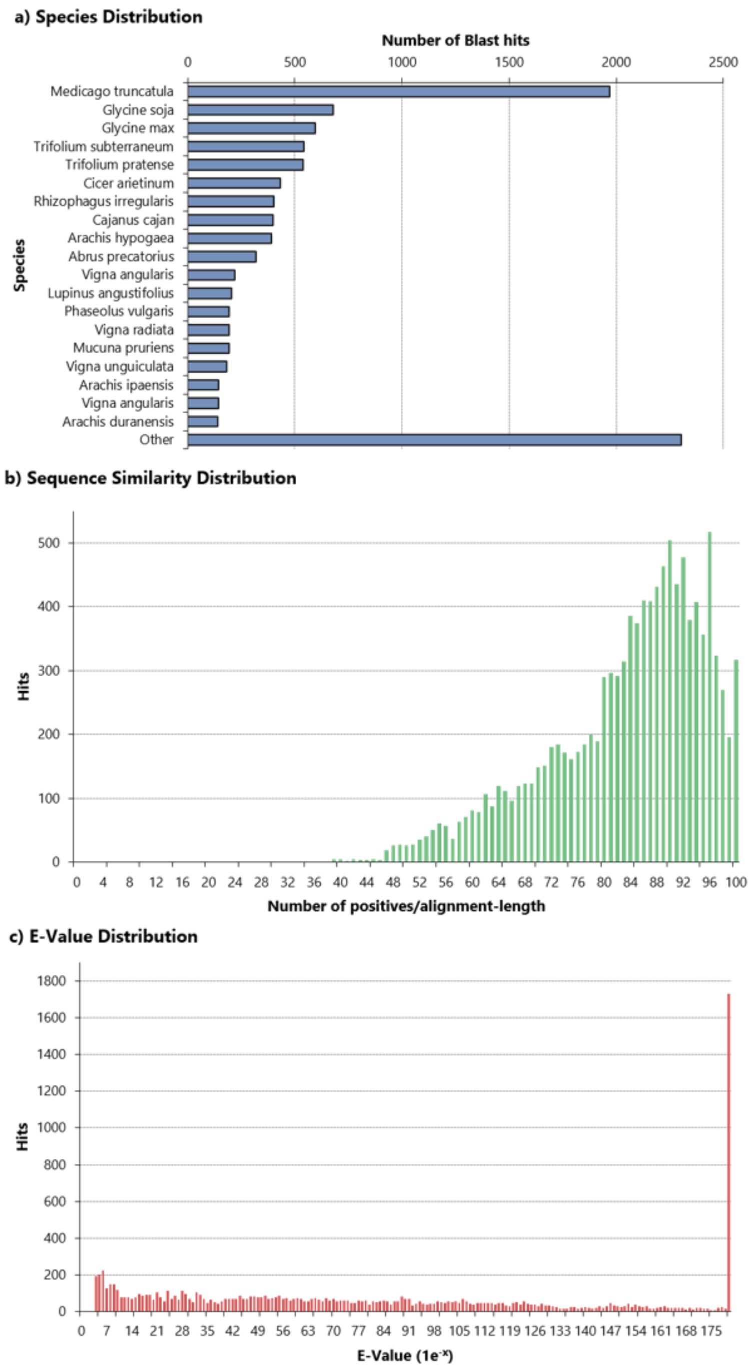

Supplementary Fig. S8. BLAST sequencing results for DE transcripts of VFA treatment from de novo RNAseq analysis, showing results for root tissue only; DE transcripts were processed in Blast2GO (Conesa *et al.*, 2005; Gotz *et al.*, 2008) and using the pipeline transcripts for BLAST result against NCBI's non-redundant NR database (Pruitt *et al.*, 2005) with project results as follows; a) Species distribution of number of BLAST hits; b) Sequence Similarity Distribution for hits against alignment length; c) E-Value Distribution of hits.

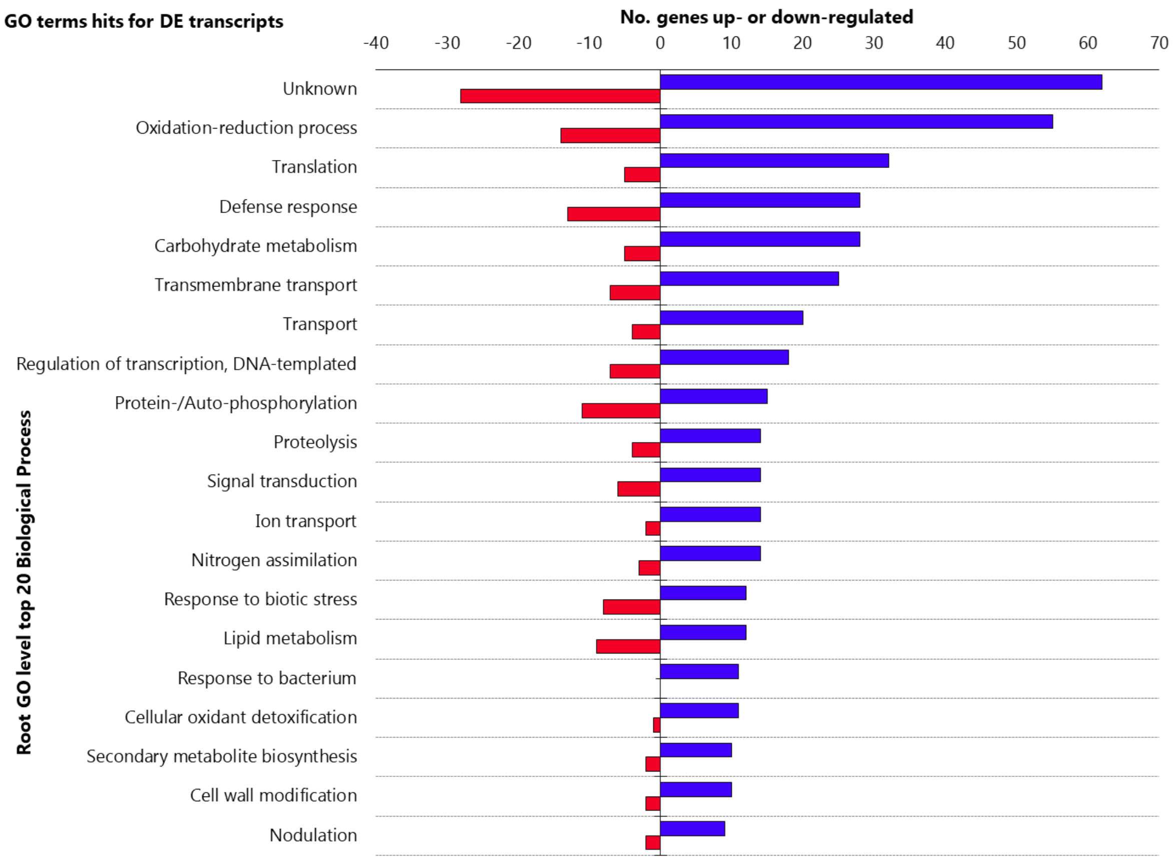

Supplementary Fig. S9. 'Biological Process' GO term hits for individual DE transcripts from VFA treatment of *Medicago sativa* roots; GO terms were pulled using Blast2GO programme mapping and ran with the EMBL-EBI InterPro library (Mitchell *et al.*, 2019), with manual addition from QuickGO (Binns *et al.*, 2009) and UniProt (The UniProt Consortium, 2018) databases. Bars show genes upregulated (blue) or downregulated (red), indicating the number of DE transcripts with the associated GO term. Only the top 20 GO terms are shown; total GO terms associated with dataset was 142.

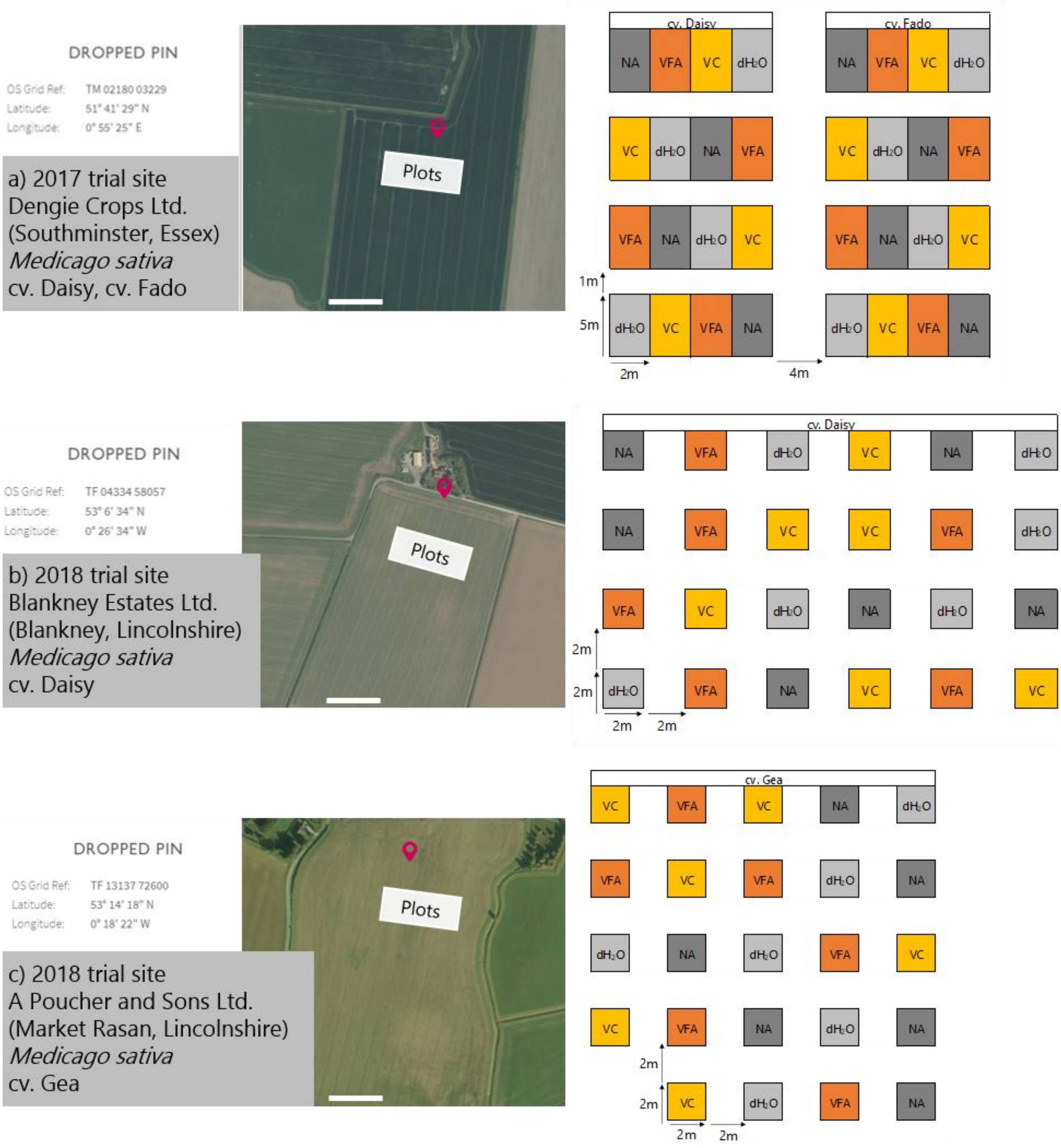

Supplementary Fig. S10. Field plots for fulvic acid trials performed in 2017 and 2018.

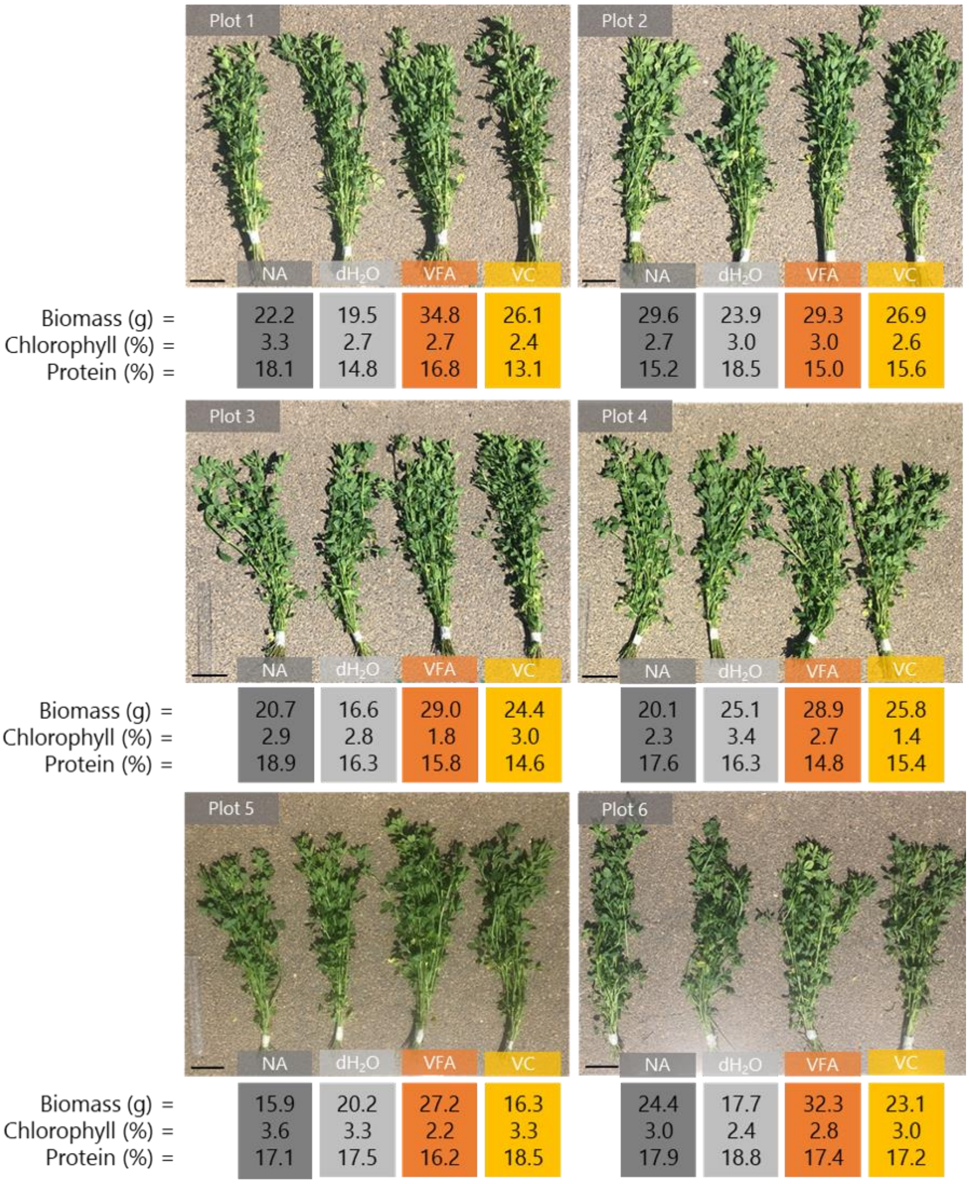

Supplementary Fig. S11: Vegetative tissue of *Medicago sativa* of first cut following fulvic acid treatment compared to a control, from field trial plots. Treatments were applied to field plots at beginning of establishment and vegetative yields were assessed before 1st cut of growing season; an area of 625 cm^2 was sampled and total vegetative tissue dried for biomass (in g), with value indicated. Treatments were; no addition (NA in dark grey); deionised water (dH2O in grey); 1 % VFA (VFA in orange); and 1 % VC (VC in yellow). Samples are from 2018 trial plots at Blankney Estates Ltd. (Blankney, Lincolnshire) for cv. Daisy. Samples were duplicated for chlorophyll and protein contents; these are provided as total dry weight %. Multiple comparisons were conducted using a one-way ANOVA Tukey test with VFA having a significant increase in biomass samples only.

References for Supplementary data

Binns D, Dimmer E, Huntley R, Barrell D, O'Donovan C, Apweiler R. 2009. QuickGO: a web-based tool for Gene Ontology searching. Bioinformatics **25**, 3045-3046.

Conesa A, Götz S, García-Gómez JM, Terol J, Talón M, Robles M. 2005. Blast2GO: A universal tool for annotation, visualization and analysis in functional genomics research. Bioinformatics **21**.

Gotz S, Garcia-Gomez JM, Terol J, Williams TD, Nagaraj SH, Nueda MJ, Robles M, Talon M, Dopazo J, Conesa A. 2008. High-throughput functional annotation and data mining with the Blast2GO suite. Nucleic Acids Research **36**, 3420-3435.

Law CW, Chen Y, Shi W, Smyth GK. 2014. voom: Precision weights unlock linear model analysis tools for RNA-seq read counts. Genome Biology **15**, R29.

Mitchell AL, Attwood TK, Babbitt PC, *et al*. 2019. InterPro in 2019: improving coverage, classification and access to protein sequence annotations. Nucleic Acids Research **47**, D351-d360.

Powell DR. 2014. Degust (formerly DGE-Vis) : Visualize, explore and appreciate RNA-seq differential gene-expression data. Monash University, Australia: Victorian bioinformatics consortium.

Pruitt KD, Tatusova T, Maglott DR. 2005. NCBI Reference Sequence (RefSeq): a curated non-redundant sequence database of genomes, transcripts and proteins. Nucleic Acids Research **33**, D501-D504.

The UniProt Consortium. 2018. UniProt: a worldwide hub of protein knowledge. Nucleic Acids Research **47**, D506-D515.
